# Differentiated prevention and care to reduce the risk of HIV acquisition and transmission among female sex workers in Zimbabwe: study protocol for the ‘AMETHIST’ cluster randomised trial

**DOI:** 10.1186/s13063-022-06119-w

**Published:** 2022-03-12

**Authors:** Frances M. Cowan, Fortunate Machingura, Sungai T. Chabata, M. Sanni Ali, Joanna Busza, Richard Steen, Nicola Desmond, Maryam Shahmanesh, Paul Revill, Amon Mpofu, Raymond Yekeye, Owen Mugurungi, Andrew N. Phillips, James R. Hargreaves

**Affiliations:** 1 Department of International Public Health, Liverpool School of Tropical Medicine, Pembroke Place, Liverpool, United Kingdom; 2 Centre for Sexual Health Zimbabwe, 4 Bath Road, Belgravia, Harare, Zimbabwe; 3 Public Health, Environments and Society, London School of Hygiene and Tropical Medicine, Keppel Street, London, United Kingdom; 4 Institute for Global Health, University College London, Gower Street, London, United Kingdom; 5 Centre for Health Economics, University of York, York, United Kingdom; 6 National AIDS Council Zimbabwe, Harare, Zimbabwe; 7 Ministry of Health and Child Care Zimbabwe, Harare, Zimbabwe

**Keywords:** Effectiveness, Hidden population, Pragmatic trials, Randomised control trial, Respondent driven sampling, Sex workers

## Abstract

**Background:**

Female sex workers (FSW) in sub-Saharan Africa are disproportionately affected by HIV and are critical to engage in HIV prevention, testing and care services. We describe the design of our evaluation of the ‘AMETHIST’ intervention, nested within a nationally-scaled programme for FSW in Zimbabwe. We hypothesise that the implementation of this intervention will result in a reduction in the risk of HIV transmission within sex work.

**Methods:**

The AMETHIST intervention (Adapted Microplanning to Eliminate Transmission of HIV in Sex Transactions) is a risk-differentiated intervention for FSW, centred around the implementation of microplanning and self-help groups. It is designed to support uptake of, and adherence to, HIV prevention, testing and treatment behaviours among FSW. Twenty-two towns in Zimbabwe were randomised to receive either the Sisters programme (usual care) or the Sisters programme plus AMETHIST. The composite primary outcome is defined as the proportion of all FSW who are at risk of either HIV acquisition (HIV-negative and not fully protected by prevention interventions) or of HIV transmission (HIV-positive, not virally suppressed and not practicing consistent condom use). The outcome will be assessed after 2 years of intervention delivery in a respondent-driven sampling survey (total *n* = 4400; *n* = 200 FSW recruited at each site). Primary analysis will use the ‘RDS-II’ method to estimate cluster summaries and will adapt Hayes and Moulton’s ‘2-step’ method produce adjusted effect estimates. An in-depth process evaluation guided by our project trajectory will be undertaken.

**Discussion:**

Innovative pragmatic trials are needed to generate evidence on effectiveness of combination interventions in HIV prevention and treatment in different contexts. We describe the design and analysis of such a study.

**Trial registration:**

Pan African Clinical Trials Registry PACTR202007818077777. Registered on 2 July 2020.

**Supplementary Information:**

The online version contains supplementary material available at 10.1186/s13063-022-06119-w.

## Background

Effective tools for preventing and treating HIV are now widely availabl e[1]. Yet, globally, gaps in implementation remain especially among vulnerable populations [1, 2]. Eastern and Southern Africa is home to approximately half of all people living with HIV and just under half of all HIV-related deaths in 2019 [3]. Only 59% of those living with HIV in the region are virally suppressed [3–5]. Female sex workers (FSW) make up a small proportion of the population [6, 7] but bear a disproportionate burden of infection. The risk of HIV acquisition is up to 21 times higher for FSW than non-sex-working women aged 15–49 years [4, 8]. Further, sex work is directly or indirectly a cause of a high proportion (40–80%) of all new infections [4–9]. Implementing strategies that improve access, uptake and effective use over time of HIV prevention and treatment tools among FSW is needed urgently.

UNAIDS/WHO recommend targeted sexual and reproductive health services (including HIV related) for FSW, supported by peer-based community outreach. However, the optimal delivery model for such services in southern African contexts remains unclear. Effectiveness studies, especially randomised controlled trials (RCT), remain rare. In Zimbabwe, we previously conducted a cluster-RCT of an intervention comprising enhanced access to regular HIV testing, on-site initiation of ART, access to pre-exposure prophylaxis (PrEP), adherence interventions and intensified community mobilisation. The intervention improved rates of HIV diagnosis and treatment initiation. Viral suppression improved in both arms of the trial, but there was no significant difference in the proportion of all FSW with unsuppressed HIV infection between the arms [10].

Building on our previous work we have developed ‘AMETHIST’ (‘Adapted Microplanning: Eliminating Transmissible HIV In Sex Transactions’), a new intervention package with a greater focus on HIV prevention to complement the existing focus on HIV testing and treatment, and sexual and reproductive health more broadly. We hypothesise that this theory-based intervention, combining microplanning and self-help groups (SHG), can raise uptake and adherence to HIV prevention and treatment tools among FSW and thereby achieve our goal of reducing the proportion of FSW at risk of acquiring or transmitting HIV infection.

Here we formally describe the ‘AMETHIST’ intervention and its intended mechanisms of action and describe the design and analysis strategy for a pragmatic cluster randomised trial to determine the impact of the intervention on HIV transmission risk among sex workers.

### The AMETHIST interventions and its mechanism of action

A structured description of the AMETHIST intervention is shown in Table 1. It incorporates key elements of both the TIDIER framework [11] (elements 1–9, first column, Table 1) for intervention description and replication, and the Proctor framework for specifying implementation strategies [12]. Elements 10 and 11 of TIDIER relate to modifications and fidelity of intervention delivery in practice and so are more suitably reported at the end of the trial and therefore excluded here.

**Table 1:**
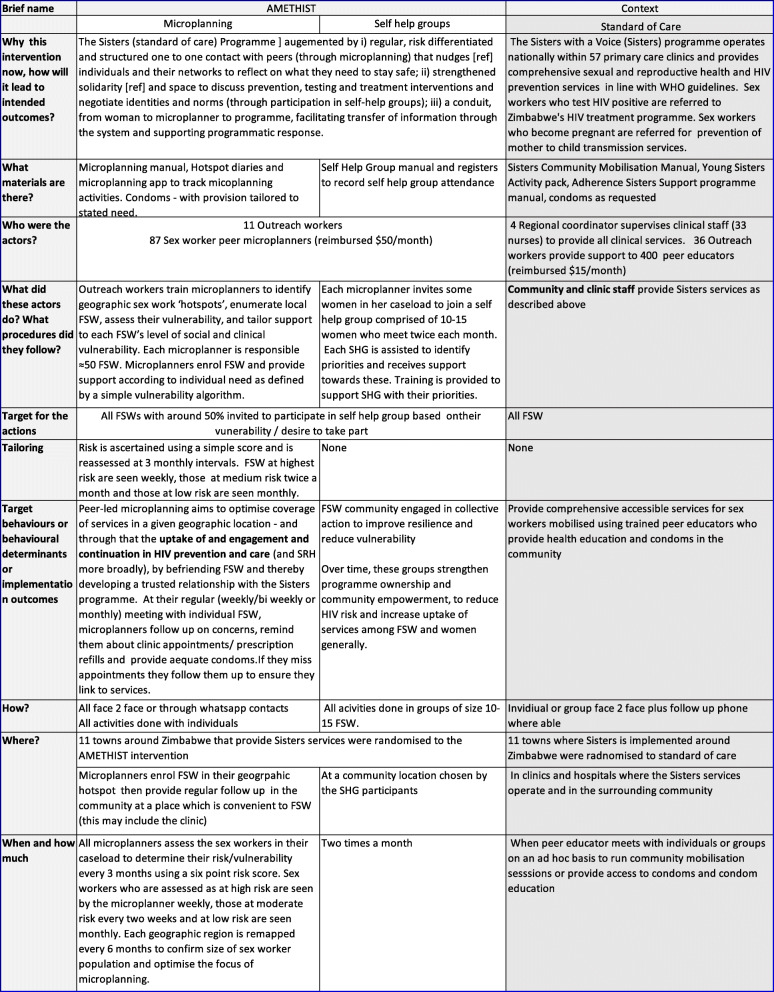
Structured description of the AMETHIST intervention and Standard of Care within which this intervention is set, drawing on TIDIER and Proctor frameworks

### Standard of Care: the Sisters with a Voice programme

The Sisters with a Voice (‘Sisters’) programme was established in Zimbabwe in 2009 on behalf of the Ministry of Health and Child Care (MoHCC) and the National AIDS Council (Table 1) [13]. It operates nationally within 57 primary care clinics and provides services in line with WHO guidelines [14]. Sisters provides free condoms and contraception, provider-initiated HIV testing and counselling, HIV self-testing and counselling (and secondary distribution of self-test kits for partners), syndromic management of sexually transmitted infections (STIs), health education and legal advice supported by a network of peer educators. Additionally, clinics provide long-acting reversible contraception (implants), referral for cervical cancer screening and on-site access to PrEP. PrEP is available as part of national roll-out across all 22 sites participating in the trial. Stock of PrEP is coordinated and monitored nationally by MoHCC and at sites by the clinic manager. The programme is supported by over 400 sex worker peer educators trained to provide basic information and undertake condom distribution to peer FSWs; they earn $15USD *per month* as stipend. They mobilise FSW to attend clinical services and encourage uptake of HIV testing and support referral of FSW for antiretroviral therapy (ART) for those who test positive for HIV to government services for HIV care/ART initiation. Outreach worker (ORW) supervisors who are salaried social workers, meet with the peer educators at each site once a month as a group to discuss issues that have arisen. Programme data are collected electronically in real-time.

### AMETHIST intervention

**Fig. 1:**
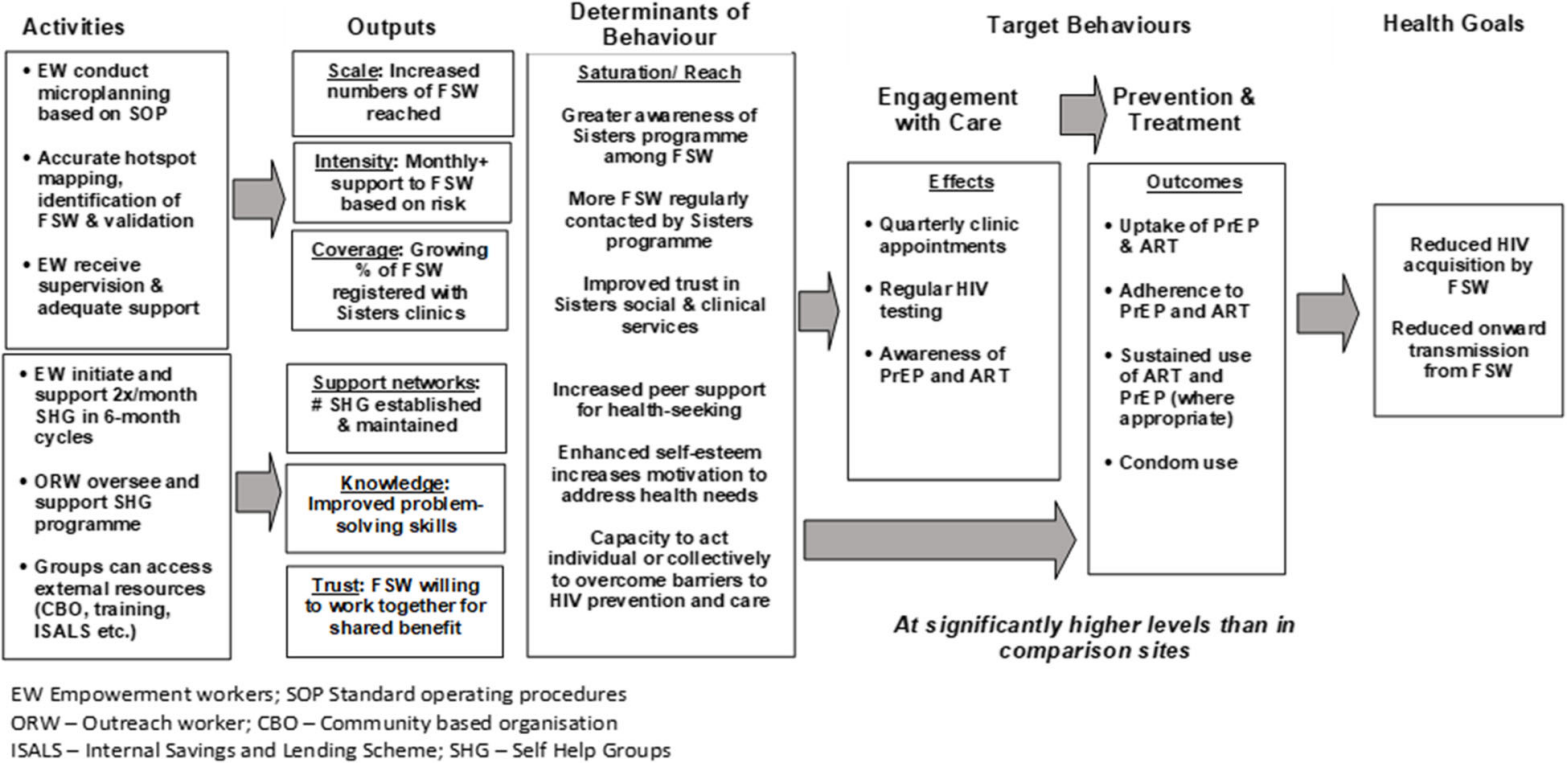
Project trajectory outlining the intended mechanisms of action of the AMETHIST intervention

In the AMETHIST intervention, we will introduce ‘microplanning’ and establish SHG, layered on top of the existing Sisters programme (Table 1). Through these components, the AMETHIST intervention seeks to strengthen FSW self-esteem, social support and group capacity [15, 16] as well as their motivation and opportunity to access services [17]. Through this focus on peer empowerment and community self-efficacy, the intervention seeks to enhance the capability of all FSW to overcome entrenched barriers to the adoption of behaviours that decrease HIV transmission (Fig. 1).

Both components of the AMETHIST intervention occur outside of formal clinical settings and are delivered in such a way as to be ‘HIV status neutral’. While the specific technologies and pathways of care for prevention and treatment differ, there are important similarities. Social and structural factors such as stigma, lack of social cohesion and the low socioeconomic status of female sex workers act as barriers to the uptake and effective use over time of both prevention and treatment tools. Condom use offers ‘dual protection’ from both HIV transmission and acquisition, and consistent condom use is strongly recommended for all FSW. Antiretroviral medications prescribed to be taken daily, albeit in different formulations and under different clinical supervision approaches, form a component of both treatment (as ART) and prevention (in the form of PrEP). The AMETHIST intervention seeks to reduce the risk of both HIV acquisition and transmission among FSW by targeting these common drivers, thereby reducing the role of commercial sex work in HIV transmission more broadly. Furthermore, a ‘HIV status neutral’ approach reduces a source of tension and mistrust between sex workers and retains a focus on the priorities and concerns they share as a community.

#### Microplanning

Peer-led microplanning aims to optimise programme coverage and support prevention and care uptake [18]. While it has been successful in India [19, 20] as a critical element of HIV prevention programming, it has not been widely implemented or evaluated in Africa. The sex workers who undertake the microplanning are called empowerment workers (EWs). EWs are FSW peer educators who additionally have a specified caseload of sex workers for whom they are responsible (*n* = 50–80), undergo additional training in data collection and interpretation and are expected to devote more time to microplanning than standard peer educators. Unlike the peer educators in the standard of care group, EWs are trained to identify geographic sex work ‘hotspots’, enumerate FSW working within those hotspots, assess their vulnerability and tailor support to each FSW’s level of social and clinical vulnerability. Hotspots are geographical locations where FSWs work and are places where high HIV risk behaviour may take place (or is negotiated) and includes streets, truck-stops, bars, lodges, hotels and brothels. Hotspots will be identified by EWs working in a geographic area. Care will be taken to ensure EWs have non-overlapping hotspots.

Microplanning incorporates a risk-differentiated approach to the support EWs provide to other FSW. EWs assess FSW’s vulnerability to HIV acquisition/transmission risk, and this guides the intensity with which microplanning is implemented. This vulnerability is assessed in the field using a simple algorithm based on age, duration in sex work, client numbers, condom use consistency, alcohol use and experience of violence (see Appendix Table A1). EW reassesses the risk of their case load at 3-monthly intervals (Table 1). FSW assessed as being at high risk will be followed up weekly, those at medium risk twice a month and those at low risk monthly. At each microplanning contact, EW provide support to individual FSW guided by their tracking data (e.g. revisiting previous discussions, reminding them about upcoming clinic appointments or ensuring they have an adequate condom supply) and remind women about clinic appointments. The microplanning process thereby generates data to inform subsequent contacts. EWs update the data, help analyse it, and meet with ORW supervisors weekly to plan whom to see each week and topics for discussion. Each EW earns a stipend of $50 USD per month. ORWs supervise empowerment workers across multiple hotspots in their respective sites. Each ORW has a caseload of 7-10 EW. Geographic hotspots will be remapped every 6 months and the size of the sex work population re-estimated so that the rate of programme coverage can be monitored.

#### Self-help groups

Self-help groups aim to build social cohesion and community empowerment. Each EW invites some of the FSW in her microplanning caseload to join a SHG comprised of 10–15 women who meet twice each month. FSW are invited to participate in SHG based on their level of vulnerability and their willingness/ability to attend meetings regularly. The EW facilitates the SHG, initially with the support of an ORW. FSW in the group receive training on how to participate, keep a register, manage money (including how to budget, save, open and manage a bank account) and keep minutes if required. Each SHG is assisted to identify priorities and take relevant action. Training is provided by the programme to support SHG with addressing their priorities. Over time, these groups strengthen programme ownership and community empowerment, to reduce HIV vulnerability and increase uptake of services among FSW [15, 21]. Although not all FSW will join the initial SHG, they provide a model of community leadership that goes beyond Sisters’ standard model of community mobilisation that relies on health-related workshops with a less interactive or sustained format.

### Process evaluation and costing

We will undertake a nested process evaluation which will draw on both quantitative programme data and qualitative assessments to monitor delivery and uptake of each programme component over time, examining ease of delivery, varying rates of participation by FSW and their experiences, perceptions and satisfaction levels. We do not describe this data collection in any further detail here but provide details in the trial protocol (see Appendix A2).

We will also determine the cost of the intervention components and the cost-effectiveness of the AMETHIST intervention, taking into account the potential reduction in transmissions via selling sex due to the reduction in the proportion of FSW who are at risk of either HIV transmission or acquisition. For this, we will use an existing individual-based dynamic stochastic model to predict the potential impact of this intervention on HIV incidence in the population of Zimbabwe. These methods are not discussed further here.

## Methods

### Trial design

The trial design is a cluster randomised controlled trial.

### Cluster definition and selection

A cluster is defined as the FSW population working in the geographic location (usually a town or business centre) where there is a government health clinic providing dedicated FSW services through the Sisters programme. Trial sites were purposively selected to be reflective of a range of settings, of adequate size to ensure participation of between 175 and 475 FSW annually (based on 2017 programme data) (mean 314; 50% seen for first time) and located at geographic spacing sufficient to ensure that the risk of contamination/spill-over of intervention effect between study clusters through FSW mobility and migration will be minimised (see Appendix Fig. A1).

### Randomisation

Twenty-two sites have been randomised (1:1) to receive the AMETHIST intervention in addition to standard of care or to continue with the standard of care alone. Randomisation was conducted in January 2019 at a public meeting with key stakeholders, MoHCC, district representatives and representatives of female sex worker community from the 22 sites. It was not possible to blind intervention staff or beneficiaries of services to the intervention allocation.

To minimise baseline imbalance between arms, restricted randomisation was used [22]. Restriction factors included province, number of FSW seen in the Sisters programme in 2017, mean age of first-time attenders, proportion of FSW attending the programme who were aged under 20 years of age, proportion of all attendees aware of HIV status, proportion of all HIV-positive attendees on ART and mean number of visits by attendees. Restricted randomisation resulted in a good balance of restriction factors between treatment arms (see Appendix Table A2). Restricted randomisation generated a list of 705,432 potential combinations from which 999 with the closest balance between arms were selected. Non-investigator attendees were invited to withdraw numbers from a bag to see which one of the 999 combinations was selected.

### Primary endpoint

**Fig. 2:**
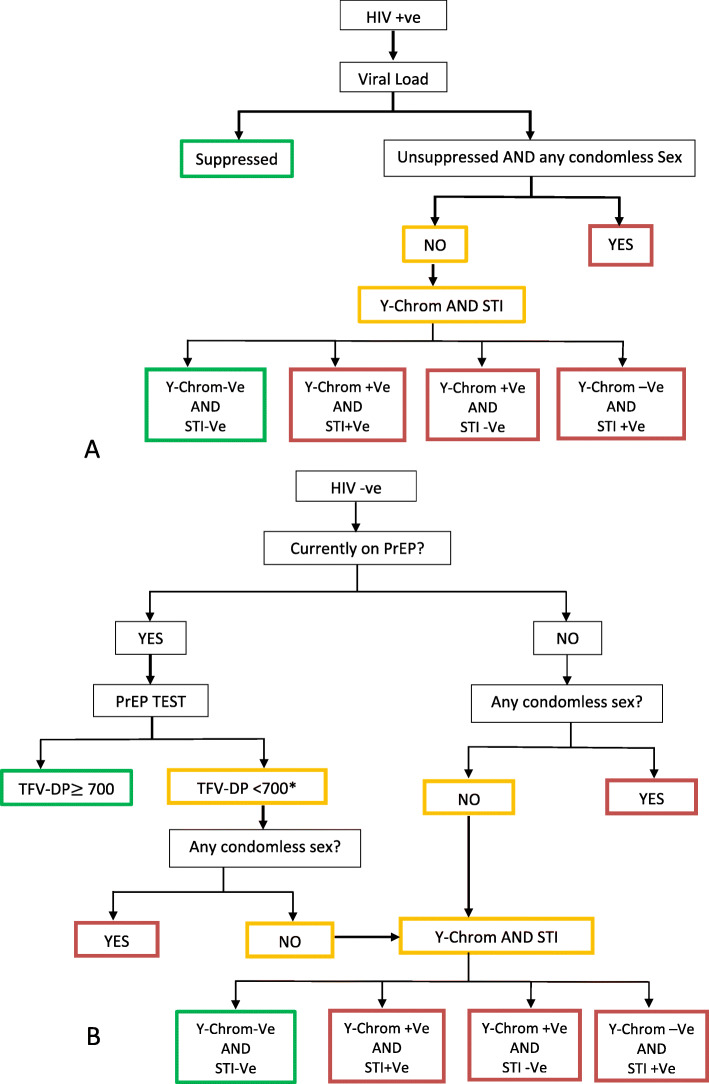
Algorithm for the primary outcome: risk of HIV transmission among HIV positives. (**a**) OR risk of acquisition among HIV negatives (**b**). Y-chromosome test (Y-Chrom), GC (*Neisseria gonorrhoea* test), PrEP (pre-exposure prophylaxis), positive (+ve) and negative (−ve). FSW is at risk of transmission (red) or not at risk of transmission (green), yellow means additional tests are needed to classify them as ‘at risk’ or ‘not at risk’ of transmission/acquisition. Several questions were used to ascertain consistent condom (any condomless sex) (see Appendix Table A3). Asterisk indicates the following: in a sensitivity analysis, 350 fmol/DBS punch threshold will be used to classify FSW currently on PrEP as high vs low PrEP adherence

The primary endpoint of the trial is a composite endpoint intended to capture the proportion of all FSW who are at risk of either HIV acquisition or HIV transmission as measured in a cross-sectional survey after 2 years of implementation delivery. Figure 2 shows how FSW primary endpoint status will be assessed (see also Appendix Table A4). At endline, all participating women will be identified with a binary endpoint measure: as either at risk of acquisition/transmission (red) or not at risk of acquisition/transmission (green). This means that both groups will include FSW who are both HIV positive (Fig. 2a) and HIV negative (Fig. 2b).

The determination of the primary endpoint incorporates data on four ‘domains’. First, their HIV status based on laboratory test at endline. Second, HIV-positive women are identified as either virally suppressed (< 1000 copies/mL) or not, based on viral load measurements at endline. Third, HIV-negative women are identified as either being on oral PrEP, or not. This is done first through self-report (where possible cross verified with programme data), and then, for those who report that they are currently taking PrEP, through requesting and analysing a blood sample for PrEP (tenofovir diphosphate) levels. Only those both reporting and having biological evidence of the presence of PrEP will be considered as ‘on PrEP’ (Fig. 2). Fourth and finally, among both HIV-positive and HIV-negative women, women will be classified as having had recent (in the past month) condomless sex unless she reports otherwise and there is not biological evidence to contradict this. This evidence will incorporate three types of information: first, women will be asked to self-report if they have had any condomless sex in the past month or not (see Appendix Table A3). For those who report no condomless sex, we will ask if they are willing to provide a sample for detection of Y chromosome, a biological marker of unprotected sexual activity with male partners [23]. In addition, all women will be offered testing for STIs including active syphilis, *Chlamydia trachomatis*, *Neisseria gonorrhoea* and *Trichomonas vaginalis*. We will consider women as having been validated as having had no recent condomless sex if she reports no condomless sex and has no evidence of vaginal *Neisseria gonorrhoea* or y chromosome in her vaginal sample (Fig. 2). Relevant data collection, questionnaire and laboratory methods are described in more detail below.

The trial protocol also pre-specifies a range of secondary outcomes summarised in Table 2.

**Table 2:** Summary of secondary outcomes

1) Proportion of FSWs at risk of HIV transmission among HIV positives	
2) Proportion of FSWs at risk of HIV acquisition among HIV negatives	
3) Proportion of HIV-infected women who are infectious (viral load > 1000copies/μL)	
4) Proportion of FSWs reporting always using condoms with clients in last month who have laboratory evidence of STI	
5) Proportion of FSWs reporting always using condoms with clients in last month who have evidence of Y chromosome in vaginal specimen	
6) Proportion of FSW who have evidence of	
a. HIV testing in the last 6 months (among those now self-reporting HIV positivity)	
b. Having attended the Sisters clinic in the last 3 months	
c. Pre-exposure prophylaxis for HIV negatives	
d. Antiretroviral therapy for HIV positives	
7) Proportion of all FSWs who know HIV status (i.e. are diagnosed HIV positive or were tested HIV negative in last 6 months)	
8) Proportion of those taking ART who have (viral load > 1000 copies/μL) who have drug resistance	

### Inclusion and exclusion criteria for outcome assessment

Women are eligible for outcome assessment if they are aged 18 or older, currently working as a sex worker (has exchanged sex for money in the past 30 days) and living or working in the study site (for at least 1 month).

### Data collection: population sampling

The trial outcome will be assessed after 24 months of intervention using a respondent driven sampling (RDS) survey in each of the trial clusters. Initial ‘seed’ women who meet the study inclusion criteria will be selected after mapping sex work in that location [24]. Selection as a seed will be independent of whether they have accessed the Sisters programme. Each seed will start a recruitment ‘chain’ by recruiting up to two peers into the survey. Each subsequent respondent will be further provided with coupons to give to up to two peers to refer them into the study. Participants will receive financial compensation for completing the survey themselves, as well as for each of their recruits who participates. Recruitment will stop when approximately 200 women have been recruited in a given site (see sample size justification below). Ethical considerations including informed consent is described later and also in the Appendix (see Appendix A2).

### Data collection: questionnaires

Questionnaire data will be collected through self-administration directly onto tablet computers using an audio computer assisted survey instrument (ACASI) in part to minimise reporting bias. Trained research assistants will assist each FSW to log-in correctly and understand the use of the ACASI. The questionnaire will include questions on demographics, sex work, sexual behaviour and condom use, HIV testing history, ART use (including specific drug regimen), stigma, experience of violence, quality of life, mental health, substance use, general health, relationships with other sex workers and use of sexual and reproductive health services. We will collect data to determine personal network size for RDS adjustment: we will ask how many females sex workers the participant knows who are aged over 18, live at the site, who she has seen in the last month and who she would consider recruiting to the study. In addition, we will collect information from consenting women to allow us to link questionnaire data to Sisters programme data.

### Data collection: sample collection and laboratory analysis

All women will have a finger prick sample collected for testing for HIV infection and syphilis on site as point of care tests. Samples for HIV will be tested according to the Zimbabwe National HIV testing algorithm with samples tested in series. The syphilis sample will be tested using *DPP*® *Syphilis Screen* & *Confirm* Assay (Chembio Diagnostic Systems, Inc. New York, USA), a near patient test that tests for both Rapid Plasma Reagin (RPR) and Treponema Pallidum Haemagglutination Assay (TPHA) in a single test in 20 min. The results of HIV and Syphilis rapid tests will be returned to all participants on site. All women will also have two dried blood spot samples (DBS) collected for HIV viral load testing, LAg avidity assay testing (for incorporation into a recent infection testing algorithm) [25], drug resistance testing, ART level testing and PrEP level testing as indicated. The results of viral load tests will be made available to women within 4 weeks of the survey. DBS samples will be shipped to University of Cape Town for ART testing to assess presence of ART in FSW with evidence of recent HIV infection and a viral load < 1000 copies/ml and for Tenofovir-diphosphate (TDF-DP) in HIV-negative women reporting use of PrEP (PrEP adherence is defined as ‘*high*’ if Tenofovir-diphosphate (TDF-DP) ≥ 700 fmol/dDBS punch, ‘*low*’ if < 350 fmol/punch, and medium if 700 < TDF-DP ≤ 350 fmol/punch) [26]. For our primary outcome, medium and low levels (< 700 fmol/punch) will be pooled into one category as ‘low’ (Fig. 2). All laboratory staff undertaking laboratory analyses will be blind to trial arm.

In addition, all participants will be asked to provide a self-administered vaginal swab to be tested for STIs *Neisseria gonorrhoea*, *Chlamydia trachomatis* and *Trichomonas vaginalis*. All vaginal samples will be transported within 48 h at 4 °C. Samples will be tested by the Newlands Clinic using Allplex™ STI Essential Assay Q (Seegene Inc Seoul, Republic of Korea). The results of STI tests will be made available to women through the Sisters clinic within 4 weeks of the survey, and free treatment will be made available and contact tracing of sexual partners offered. All laboratory staff undertaking laboratory analyses will be blind to trial arm.

### Sample size and power considerations

The sample size of the trial was based on pragmatic considerations as well as statistical power. We chose to include 22 clusters and to recruit approximately 200 FSW in each cluster. It was not practically possible to include more than 22 clusters from across the country in the trial, and prior work has suggested that it should be possible to recruit 200 FSW in each included cluster, but in some clusters not more than this. We used restricted randomisation to minimise differences by arm. We used the approach of Hayes and Bennett for cluster randomised trials [27] to consider sample size and power. We estimated that 30% of FSW would meet our primary outcome definition. In a 2016 [10] survey, 22% of FSW were at risk of acquisition/transmission of HIV by a measure that did not include verification of self-reported consistent condom use, while in another 2017 survey 28% of FSW were at risk of acquisition/transmission of HIV using the same definition (PrEP was not available at these sites at the time of the survey). In our previous trial, the coefficient of variation between clusters (*k*) was 0.17 so we estimated sample size using *k* of 0.2 and 0.25. In most scenarios, we estimate 90% power to detect a 30% difference in the proportion of FSW who are at risk of HIV acquisition/transmission between the intervention and control arms (see Appendix Table A5). If *k* is 0.25, we have 78% power to detect a 30% difference and over 99% power to detect a 50% difference.

### Statistical analysis

Our approach is based on our previously published approach to the analysis of data from the SAPPH-IRe trial [10, 28]. One substantive difference between the trials is that in the current AMETHIST trial, we were unable to conduct RDS surveys at baseline in any of the 22 clusters because funding for the AMETHIST intervention and its evaluation were secured separately; the intervention was started prior to research funding being finalised.

### Study profile

We will describe the characteristics of clusters recruited to each arm of the study using available programmatic data. Cluster drop-out is not expected but might occur if political or community acceptance for the research protocol is compromised during the trial duration. We will produce descriptive statistics to assess whether there was balance across the two arms in key socio-demographic and potentially confounding variables at the endline RDS survey to be conducted between July and October 2021.

For each arm of the study at endline, we will describe the range and mean size of the sample recruited through the RDS survey in each site and construct participant recruitment trees. We will describe by arm the range and cluster-mean of the number of women who do not recruit two participants and their understanding of the reasons for this. We will construct a trial profile diagram in line with CONSORT principles but adapted for our specific situation.

### RDS diagnostics

We will conduct recommended RDS diagnostics for each site [29] and report our findings according to STROBE guidelines for the reporting of RDS surveys [30]. As in SAPPH-IRe, the community mobilisation activities might plausibly change the structure of the FSW social networks in the intervention sites. Consequently, the RDS sampling process that runs over these networks might be biased by trial arm. To investigate these potential biases we will (1) compare self-reported network degree in the intervention versus usual care sites, (2) compare homophily/similarity between FSW and their personal networks, and between recruiters/recruitees and (3) examine whether the place of recruitment, relationship between recruiter/recruitee and motivation for recruitment differ between arms and compare time to recruit between arms.

### Analysis principles

Our primary analysis will compare FSW recruited in each community without considering direct contact with the intervention components (i.e. an ‘intention to treat’ approach). Data from individual FSW will be summarised for each cluster, and we will express the intervention effect using a prevalence ratio, prevalence difference and associated 95% confidence intervals.

We will calculate cluster summaries using the RDS-II methodology [31]. For unadjusted analysis, we will fit a linear regression model on the RDS-weighted cluster summaries, with a treatment dummy. We will use the *p*-value and confidence intervals for the coefficient of the treatment dummy.

Adjusted analysis will use the ‘two step’ method of Hayes and Moulton [32]. Age is specified a priori and will be included as a potential confounder that could affect the outcome. We will also adjust for other potential confounders that appear imbalanced by arm between survey participants recruited to the endline RDS surveys. Imbalance will be assessed using ‘eyeballing’ and expert opinion. Decisions about factors to be adjusted for will be made prior to the analysts having access to the complete primary outcome data. We will not adjust for any characteristics that may have been influenced by the intervention. To generate adjusted and RDS-II-weighted analysis, step 1, we fit a logistic regression model for each site with the primary outcome as the dependent variable, age and other variables as independent variables. This model will be used to ‘predict’ outcomes for each woman. The predicted values will be multiplied by the inverse of the degree (RDS-II) to account for the sampling design, normalised for the cluster, and cluster summaries calculated. Step 2, we will divide the cluster summaries used in the unadjusted analysis (accounting for RDS only) by the adjusted cluster summaries from step 1 to generate ‘residuals’. We will calculate the prevalence ratio using the regression model described above with the residuals as the dependent variable. We will calculate the ‘residual’ risk difference (RD) by subtracting the predicted prevalence from the RDS-II weighted observed prevalence in each cluster. We will fit a linear regression model on the residuals to calculate the adjusted RD and 95% confidence intervals.

### Sensitivity analysis

To investigate robustness of our primary effect estimate, we will conduct sensitivity analyses in which we (1) re-calculate the primary effect only in FSW who enrolled in AMETHIST intervention compared with individually matched controls, (2) re-calculate the primary effect only in FSW who have been in contact with the Sisters programme, (3) re-calculate the primary effect estimate using cluster-summaries unadjusted for RDS-II methodology (without weighting) and (4) re-calculate the estimated effect using the ‘Successive Sampling’ estimator (‘RDS-SS’), designed to avoid the with-replacement assumption in the random walk model. RDS-SS requires estimates of the population size of FSW to already be available [33]. We will also conduct RDS-SS analyses for a range of possible population sizes [33] and assess whether findings differ using these estimates.

## Discussion

We describe the intervention, rationale, trial design and analysis plan for a pragmatic cluster randomised trial conducted within the Sisters programme in Zimbabwe of a complex intervention combining microplanning and self-help groups to support the uptake of and adherence to HIV prevention and treatment interventions for female sex workers.

We have adapted approaches found to be effective in other contexts in building community capacity to reduce vulnerability to, and improve management of, HIV infection. First, microplanning formalises peer outreach activities into a series of specified activities that engage FSW at each stage, i.e. mapping hotspots, identifying local sex workers, conducting routine risk assessments and collecting and analysing data with support from outreach workers [34]. This process allows for greater flexibility and local responsiveness of HIV programmes delivered at scale, and further consolidates FSWs leadership and ownership over the programme [35]. Second, the empowerment workers, in the AMETHIST intervention, are given further responsibility for bringing groups of 10–15 FSW together into self-help groups where they can discuss shared concerns, provide group support and eventually take collective action to address identified priorities. Self-help groups have been shown to empower poor women from low and middle income countries financially, socially and politically [36]. Qualitative studies suggest that self-help groups work through building social cohesion and self-efficacy, which contribute to health-enhancing behavioural change at community level [37].

Both our trial design and analysis strategy have multiple strengths. AMETHIST is the first cluster randomised trial of a risk differentiated, need-based and targeted intervention for HIV prevention and treatment among female sex workers in Africa. The cluster randomised design facilitates the simultaneous estimation of the intervention impact on both HIV-negative and positive women. The research participants contributing to primary endpoint analysis will be recruited through RDS surveys within trial clusters, as in our previous ‘SAPPH-IRe’ trial. RDS reduces sampling bias and improves representativeness of ‘hard-to-reach’ populations by limiting the number of referrals any one respondent can have creating ‘deep’ rather than ‘wide’ sample networks [31]. We have outlined our RDS implementation in advance, described potential biases related to the use of RDS design in practice, attempted to collect information vital to signal whether potential biases exist, and planned sensitivity analyses to quantify their impacts on our findings.

Our design has also limitations: a longer trial would have been preferred. To minimise contamination between FSW communities/spill-over effect, we used 22 clusters, which might compromise the power of the study. However, our study is larger than previous clustered randomised trials of similar interventions in Africa [10]. We also sought to strengthen power by using restricted randomisation based on key characteristics of FSWs to maximise the chance of baseline balance.

Pragmatic trials that aim to evaluate the success and effectiveness of complex interventions pose particular challenges. One such challenge is how best to characterise the interventions under study and their hypothesised mechanism of action. This is essential so that interventions can be replicated, users of the research findings know what would be necessary to implement the intervention in other settings, and the generalisability of the findings can be considered. We combined aspects of the TIDIER and Proctor frameworks to provide a structured description of the intervention. In addition, we explicitly delineate each step through which we anticipate our intervention components will lead to measurable change, which we will test through a process evaluation conducted throughout the trial. Using mixed methods, we will formally document the intervention’s fidelity to design, feasibility of delivery and acceptability to the intended target audience as well as contextual factors that may have affected its implementation and effectiveness.

Another challenge for our trial design has been in deciding on the most appropriate primary endpoint and how this will be measured. We have proposed a composite endpoint including data on four domains that together reflect the risk of a woman (either HIV positive or negative) being involved in HIV transmission/acquisition. The use of this composite endpoint poses both conceptual and methodological challenges. Composite endpoints are commonly used in clinical trials, including in HIV [38], with the most common reason being that this can increase statistical power. However, difficulties in interpretation are recognised. The multiple outcomes included within a composite endpoint may differ in their importance to clients and providers, may differ in their absolute frequency and may experience different risk reductions as a result of the intervention, and all of these effects influence interpretation [39]. While we do not currently have available population level data among FSW data on the outcome as we will measure it in the trial, we do have similar data from previous RDS surveys, when either 22.0% [10] (no drug levels or verification of reported condom use) or 28.4% [7] (no drug levels but STI test results available) of FSW were positive for our primary outcome, which the intervention seeks to reduce. We anticipate the prevalence of the actual outcome will be higher if validation of self-reports of PrEP and condom use are included. In both scenarios, approximately twice as many women who could be beneficially affected by the intervention are HIV-negative than are HIV positive likely reflecting the stage of the epidemic in Zimbabwe where although there have been considerable gains in terms of treatment coverage, the rate of new infections has remained relatively static [40]. Optimising coverage and continuation of PrEP in AMETHIST sites has the potential to impact our primary outcome.

The key methodological challenge relates to the validity of self-reported data on sexual activity, condom use and uptake/adherence to medications. Our approach assumes that the likelihood that people will report *not* using PreP and/or *not* using condoms when they actually are doing so is low, and so we assign such people as at risk of HIV transmission on the basis of their self-reports. However, for those who report these protective behaviours, our approach is to collect additional information for these individuals to be classified as *not at risk.*

## Trial status

Protocol version 1.4 on 19 February 2020. Data collection from the sites will take place starting from October 2021 and will be completed 31 December 2021.

## Supplementary Information

**Additional file 1: Table A1.** Risk assessment tool for risk differentiated microplanning. **Table A2.** Site level Characteristics between intervention and control arms used in restricted randomisation. **Table A3.** List of questions to ascertain consistent condom use. **Table A4.** Measurement of the AMETHIST primary outcome. **Table 5A.** Power calculations. **Fig. 1A.** AMETHIST trial sites. **Appendix A1.** Ethical considerations. **Appendix A2.** AMETHIST Trial Protocol

## References

[1] Geng EH, Holmes CB, Moshabela M, Sikazwe I, Petersen ML (2020). Personalized public health: an implementation research agenda for the HIV response and beyond. PLOS Medicine.

[2] Geng E, Hargreaves J, Peterson M, Baral S (2019). Implementation research to advance the global HIV response: introduction to the JAIDS supplement. JAIDS Journal of Acquired Immune Deficiency Syndromes.

[3] HIV/AIDS;, J.U.N.P.o., UNAIDS Global AIDS Report 2020 Seizing the Moment. 2020, Joint United Nations Programme on HIV/AIDS (UNAIDS): Geneva, Switzerland.

[4] UNAIDS. Global HIV & AIDS statistics — 2019 fact sheet. Global HIV & AIDS statistics — 2019 fact sheet [webpage] 2019 2020 [cited 2020 1 June 2020]; Available from: https://www.unaids.org/en/resources/fact-sheet.

[5] UNAIDS (2019). UNAIDS Data, 2019, UNAIDS.

[6] Vandepitte J (2006). Estimates of the number of female sex workers in different regions of the world. Sexually transmitted infections.

[7] Fearon E. Estimating the population size of female sex workers in Zimbabwe: comparison of estimate obtained using different methods in twenty sites and development of a national-level estimate. J Acquir Immune Defic Syndr. 2020;85:30–8. 10.1097/QAI.0000000000002393 PMC7417013 32379082

[8] UNAIDS. UNAIDS Guidance Note on HIV and Sex Work. Geneva: UNAIDS; 2012.

[9] HIV Prevention 2020 Road Map: accelerating HIV prevention to reduce new infections by 75%. Geneva: Joint United Nations Programme on HIV/AIDS (UNAIDS); 2017.

[10] Cowan FM, Davey C, Fearon E, Mushati P, Dirawo J, Chabata S, Cambiano V, Napierala S, Hanisch D, Wong-Gruenwald R, Masuka N, Mabugo T, Hatzold K, Mugurungi O, Busza J, Phillips A, Hargreaves JR (2018). Targeted combination prevention to support female sex workers in Zimbabwe accessing and adhering to antiretrovirals for treatment and prevention of HIV (SAPPH-IRe): a cluster-randomised trial. Lancet HIV.

[11] Hoffmann TC, Glasziou PP, Boutron I, Milne R, Perera R, Moher D, Altman DG, Barbour V, Macdonald H, Johnston M, Lamb SE, Dixon-Woods M, McCulloch P, Wyatt JC, Chan AW, Michie S (2014). Better reporting of interventions: template for intervention description and replication (TIDieR) checklist and guide. BMJ : British Medical Journal.

[12] Proctor EK, Powell BJ, McMillen JC (2013). Implementation strategies: recommendations for specifying and reporting. Implementation Science.

[13] Cowan FM (2019). Strengthening the scale-up and uptake of effective interventions for sex workers for population impact in Zimbabwe. J Int AIDS Soc.

[14] Cowan FM, Davey CB, Fearon E, Mushati P, Dirawo J, Cambiano V, Napierala Mavedzenge S, Hanisch D, Wong-Gruenwald R, Chemhuru M, Masuka N, Hatzold K, Mugurungi O, Busza J, Philips AN, Hargreaves JR (2017). The HIV care cascade among female sex workers in Zimbabwe: results of a population-based survey from the sisters antiretroviral therapy programme for prevention of HIV, an integrated response (SAPPH-IRe) trial. JAIDS Journal of Acquired Immune Deficiency Syndromes.

[15] Kerrigan D, Kennedy CE, Morgan-Thomas R, Reza-Paul S, Mwangi P, Win KT, McFall A, Fonner VA, Butler J (2015). A community empowerment approach to the HIV response among sex workers: effectiveness, challenges, and considerations for implementation and scale-up. Lancet.

[16] Biradavolu MR, Burris S, George A, Jena A, Blankenship KM (2009). Can sex workers regulate police? Learning from an HIV prevention project for sex workers in southern India. Social Science & Medicine.

[17] Kerrigan D, Barrington C, Donastorg Y, Perez M, Galai N (2016). Abriendo Puertas: feasibility and effectiveness a multi-level intervention to improve HIV outcomes among female sex workers living with HIV in the Dominican Republic. AIDS Behav.

[18] Blanchard JF, et al. Concepts and strategies for scaling up focused prevention for sex workersin India. Sex Transm Infect. 2008;84(Suppl 2):19–23. 10.1136/sti.2008.033134 18799487

[19] Blanchard A (2013). Community mobilization, empowerment and HIV prevention among female sex workers in south India. BMC Public Health.

[20] Beattie TS (2014). Community mobilization and empowerment of female sex workers in Karnataka State, South India: associations with HIV and sexually transmitted infection risk. American journal of public health.

[21] Campbell C, Scott K, Nhamo M, Nyamukapa C, Madanhire C, Skovdal M, Sherr L, Gregson S (2013). Social capital and HIV competent communities: the role of community groups in managing HIV/AIDS in rural Zimbabwe. AIDS Care.

[22] Li F, Lokhnygina Y, Murray DM, Heagerty PJ, DeLong ER (2016). An evaluation of constrained randomization for the design and analysis of group-randomized trials. Statistics in Medicine.

[23] Zenilman J (2001). Detecting sperm DNA in vaginal swabs by PCR: a new bio-marker to validate recent sexual behavior and condom use. Int-J-STD-AIDS.

[24] Chiyaka T, Mushati P, Hensen B, Chabata S, Hargreaves JR, Floyd S, Birdthistle IJ, Cowan FM, Busza JR (2018). Reaching young women who sell sex: methods and results of social mapping to describe and identify young women for DREAMS impact evaluation in Zimbabwe. PLOS ONE.

[25] Rice BD, Wit M, Welty S, Risher K, Cowan FM, Murphy G, Chabata ST, Waruiru W, Magutshwa S, Motoku J, Kwaro D, Ochieng B, Reniers G, Rutherford G (2020). Can HIV recent infection surveillance help us better understand where primary prevention efforts should be targeted? Results of three pilots integrating a recent infection testing algorithm into routine programme activities in Kenya and Zimbabwe. Journal of the International AIDS Society.

[26] Anderson PL, Liu AY, Castillo-Mancilla JR, Gardner EM, Seifert SM, McHugh C, Wagner T, Campbell K, Morrow M, Ibrahim M, Buchbinder S, Bushman LR, Kiser JJ, MaWhinney S (2017). Intracellular tenofovir-diphosphate and emtricitabine-triphosphate in dried blood spots following directly observed therapy. Antimicrobial agents and chemotherapy.

[27] Hayes R, Bennett S (1999). Simple sample size calculations for cluster randomized trials. International Journal of Epidemiology.

[28] Hargreaves JR, Fearon E, Davey C, Phillips A, Cambiano V, Cowan FM (2016). Statistical design and analysis plan for an impact evaluation of an HIV treatment and prevention intervention for female sex workers in Zimbabwe: a study protocol for a cluster randomised controlled trial. Trials.

[29] Gile KJ, Johnston LG, Salganik MJ (2015). Diagnostics for respondent-driven sampling. J R Stat Soc Ser A Stat Soc.

[30] White RG, Hakim AJ, Salganik MJ, Spiller MW, Johnston LG, Kerr L, Kendall C, Drake A, Wilson D, Orroth K, Egger M, Hladik W (2015). Strengthening the Reporting of Observational Studies in Epidemiology for respondent-driven sampling studies: "STROBE-RDS" statement. Journal of clinical epidemiology.

[31] Volz E, Heckathorn DD (2008). Probability based estimation theory for respondent driven sampling. Journal of Official Statistics.

[32] Hayes JR, Moulton LH. Cluster randomised trials. Boca Raton: Chapman and Hall/CRC; 2009.

[33] Gile KJ (2011). Improved inference for respondent-driven sampling data with application to HIV prevalence estimation. Journal of the American Statistical Association.

[34] Reza-Paul S, Steen R, Maiya R, Lorway R, Wi TE, Wheeler T, Dallabetta G (2019). Sex worker community-led interventions interrupt sexually transmitted infection/human immunodeficiency virus transmission and improve human immunodeficiency virus cascade outcomes: a program review from South India. Sex Transm Dis.

[35] McClarty LM (2018). Key Programme Science lessons from an HIV prevention 'Learning Site' for sex workers in Mombasa, Kenya. Sex Transm Infect.

[36] Brody C (2015). Economic self-help group programs for improving women’s empowerment: a systematic review.

[37] Morrison J, Osrin D, Alcock G, Azad K, Bamjan J, Budhathoki B, Kuddus A, Mala MA, Manandhar D, Nkhata A, Pathak S, Phiri T, Rath S, Tripathy P, Costello A, Houweling TAJ (2019). Exploring the equity impact of a maternal and newborn health intervention: a qualitative study of participatory women’s groups in rural South Asia and Africa. International Journal for Equity in Health.

[38] Wittkop L, Smith C, Fox Z, Sabin C, Richert L, Aboulker JP, Phillips A, Chêne G, Babiker A, Thiébaut R, NEAT-WP4 (2010). Methodological issues in the use of composite endpoints in clinical trials: examples from the HIV field. Clin Trials.

[39] McCoy CE (2018). Understanding the use of composite endpoints in clinical trials. The western journal of emergency medicine.

[40] Zimbabwe population-based HIV impact assessment ZIMPHIA 2020. Harare: Ministry of Health and Child Care (MOHCC); 2020.

