# Supplementary material for: Differentiated prevention and care to reduce the risk of HIV acquisition and transmission among female sex workers in Zimbabwe: study protocol for the ‘AMETHIST’ cluster randomised trial
Source: Trials. 2022 Mar 12;23:209. doi: 10.1186/s13063-022-06119-w (PMC8917622; doi:10.1186/s13063-022-06119-w)
Supplement: Supplementary file 1 — Additional file 1: Table A1. Risk assessment tool for risk differentiated microplanning. Table A2. Site level Characteristics between intervention and control arms used in restricted randomisation. Table A3. List of questions to ascertain consistent condom use. Table A4. Measurement of the AMETHIST primary outcome. Table 5A. Power calculations. Fig. 1A. AMETHIST trial sites. Appendix A1. Ethical considerations. Appendix A2. AMETHIST Trial Protocol [file 13063_2022_6119_MOESM1_ESM.docx]

**List of Appendix:**

**Table A1:** Risk assessment tool for risk differentiated microplanning

**Table A2:** Site level Characteristics between intervention and control arms used in restricted randomisation

**Table A3:** List of questions to ascertain consistent condom use

**Table A4:** Measurement of the AMETHIST primary outcome

**Table 5A:** Power calculations

**Figure 1A** AMETHIST trial sites

## **Appendix A1:** Ethical considerations

## **Appendix A2**: AMETHIST Trial Protocol

**Table A1:** Risk assessment tool for risk differentiated microplanning

|  | **Characteristics** | **Score** |
| --- | --- | --- |
| 1 | Young (<25, ≥25) | 0:1 |
| 2 | New to sex work (< 6months) | 0:1 |
| 3 | High client numbers (>10/week) | 0:1 |
| 4 | Inconsistent condom use in the last week | 0:1 |
| 5 | Problematic drinking | 0:1 |
| 6 | Problematic violence | 0:1 |
|  | **Total score (risk)** | **0 : 1 : 2 : 3 : 4 : 5 : 6** |

* Risc score 0 = low; 1-2 = medium; 3-6= high

**Tabl1 A2:** Site level Characteristics between intervention and control arms used in restricted randomisation

| **Characteristics** | **AMETHIST sites** | **Usual Care sites** |
| --- | --- | --- |
| **Number of FSW seen in the Sisters programme in 2017** | **348** | **286** |
| **Mean age of first time attenders** | **29** | **30** |
| **% of FSW <20 years of age** | **10** | **6** |
| **% of all attendees aware of HIV status** | **73** | **80** |
| **% of all positive attendees on ART** | **52** | **50** |
| **Mean number of visits by attendees** | **2** | **2** |

**Table A3:** List of questions to ascertain consistent condom use

|  | Questions | Responses |
| --- | --- | --- |
| 1 | Did you use a condom the last time that you had vaginal sex? | Yes/No |
| 2 | In the past two weeks did you use a condom every time you had vaginal sex? | Yes/No |
| 3 | In the past month, how often did you use condoms with your steady partner? | Yes/No |
| 4 | The last time you had sex with your steady partner, did you use a condom with him? | Yes/No |
| 5 | In the past month, have there been any times you did not use condoms? | Yes/No |
| 6 | The last time you had sex with a new client, did you use a condom? | Yes/No |
| 7 | The last time you had sex with a repeat client, did you use a condom? | Yes/No |
| 8 | Thinking again about all your clients in the last month, have there been any times when you did not use condoms? | Yes/No |
| 9 | In the past two weeks did you use a condom every time you had anal sex?" | Yes/No |

**Table A4:** Measurement of the AMETHIST primary outcome

|  | **HIV positive** | | **HIV negative** | |
| --- | --- | --- | --- | --- |
|  | **Not at risk** | **At risk of transmission** | **At risk of acquisition** | **Not at risk** |
| Viral load | Suppressed | Unsuppressed | - | - |
| Oral pre-exposure prophylaxis | - | - | Self-report not on PreP  OR  (Report on PREP  AND ((refuse a sample for PrEP testing) OR (PrEP adherence defined as ‘*high’* if Tenofovir-diphosphate (TDF-DP) ≥ 700 fmol/dried blood spot (DBS) punch, and *‘low’ if* < 700 fmol/punch.)) | Report on PrEP, confirmed by drug levels indicating high PrEP adherence TFV-DP ≥ 700 fmol/dried blood spot (DBS) punch |
|  | OR | AND | AND | OR |
| Evidence of condom-less sex | Self-report no condomless sex AND  agree to a Y Chromosome test  AND  Y Chromosome negative  AND  Bacterial STI negative | Self-report any condomless sex in past 1 month  OR  (Self-report no condom-less sex AND  ((Refuse a sample for Y chromosome testing) OR (Y Chromosome positive))  OR  (Lab-positive for a bacterial STI)) | Self-report any condomless sex in past 1 month  OR  (Self-report no condom-less sex AND  ((Refuse a sample for Y Chromosome testing)  OR (Y Chromosome positive))  OR  (Lab-positive for a bacterial STI)) | Self-report no condomless sex  AND  agree to a Y Chromosome test  AND  Y Chromosome negative  AND  Bacterial STI negative |

**Table 5A:**  **Power calculations**

* most likely scenario

**Figure 1A:** AMETHIST trial sites

##
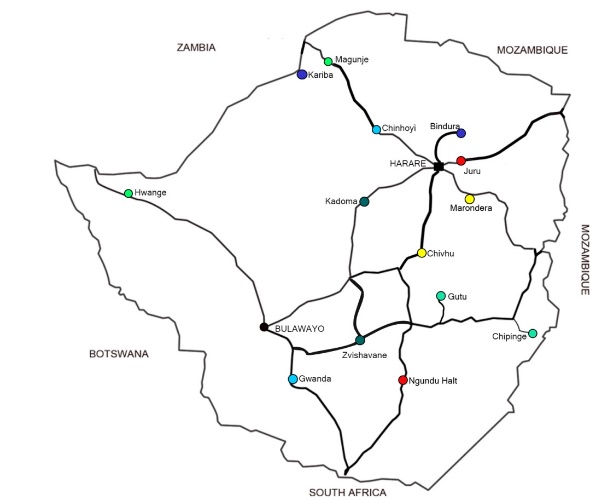


## **Appendix A1:** Ethical considerations

The trial protocol was reviewed and approved by institutional review boards at all participating institutions, including those in Zimbabwe (the Medical Research Council of Zimbabwe), Liverpool School of Tropical Medicine, University College London and London School of Hygiene and Tropical Medicine. This included, according to each IRB’s requirements, approval prior to the initiation of research, on-going adverse event monitoring, periodic review, and final study reporting. The protocol has been approved by the Ministry of Health and Child Care and the National AIDS Council who work in partnership with Sisters program to implement this study.

Of note randomisation of sites for the trial took place prior to research funding becoming available and a protocol entitle “Cluster Randomised Trial of Microplanning: An HIV Prevention and Care Differentiated Community Support Model for Female Sex Workers" - was submitted to and approved by MRCZ (MRCZ/E/206). This protocol was to randomise sites to AMETHIST intervention or standard of care and measure outcomes using programme data only. No research data were to be (or have been) collected under protocol MRCZ/E/206.

### Participant informed consent and remuneration

Survey participants will be recruited under good clinical practice by experienced and female researchers trained and certified in Ethics, Good Clinical Practice and Safeguarding. Written informed consent (Annex 3), for survey participation will be conducted. Participants will be provided with information about the research and study procedures by a trained female interviewer and will have the opportunity to ask questions as part of the informed consent process. Standard guidelines for illiterate participants will be followed and are detailed in the SOP.

Women who participate in the RDS surveys will be recruited through RDS survey techniques, which include recruiting 6-10 initial seeds at each site in both the intervention and control communities. Participants will receive financial compensation of US$5 for completing the survey themselves as well as US$2 for each of their recruits who participates. SW, health care workers and other stakeholders who are interviewed as part of process evaluation will also be recompensed with the standard US$5.

### Confidentiality

The research team ensure that all research data collected are numbered with a unique ID and not named. A link log which links the unique ID to name will be kept by the project coordinator in Harare in a locked and secure place, separate from the questionnaire/interview data. Names, addresses and other identifying information will be required at the clinic sites for follow up purposes, and will be kept separately from questionnaire and laboratory data. Only the site coordinator, data manager and principal investigator will have access to this information. Laboratory and questionnaire data will be linked using an individual's unique study ID number.

All study staff had GCP and ethics training. All people working with CeSHHAR Zimbabwe signed a confidentiality agreement; they have strict confidentiality procedures in place.

**Trial Registration**

The trial was registered at the Pan African Clinical Trial Registry (PACTR202007818077777) on 02 June 2020

### AMETHIST Trial Data and Safety Monitoring Board

A Data and Safety Monitoring Board (DSMB) was established and meets at the outset of the trial using Skype/ZOOM, and then semi-annually. The DSMB approved the overall protocol, assessments, and consent forms. The DSMB will receive all reports of adverse events, as will the IRBs overseeing this study. Meetings of the DSMB can be scheduled, as needed, to discuss and resolve AE issues. The DSMB will review semi-annually all accrued data to assess that study objectives are being met, and to ensure that benefit exceeds harm. The DSMB will see and approve the Statistical Analysis Plan prior to endline analysis.

### AMETHIST Community Advisory Board

The trial established a community advisory board (CAB) comprising one SW and one community stakeholder from each of the trial communities. The mandate of the SW Community Advisory Board is to strongly link the trial steering committee and investigators to the SW community, ensure transparent and full communication between trial and the community it serves, and to ensure that the trial research findings have a maximum impact on the communities from which the information is drawn. The CAB functions in an advisory capacity to the trial steering committee, which will be endeavoured to respond appropriately to the issues raised. The CAB assist trial staff in developing culturally and linguistically appropriate materials for study participants such as study specific fact sheets and informed consent forms.

**Appendix A2:** AMETHIST Trial Protocol – Abstracted from AMETHIST Consortium Protocol

# INVESTIGATORS AND PARTICIPATING INSTITUTIONS

## Investigators

***Table 1: Investigator profiles***

| **Role** | **Name** | **Credentials** | **Institution** | **Responsibilities** | **Email** |
| --- | --- | --- | --- | --- | --- |
| Principal Investigator | Professor Frances Cowan | MBBS, MRCP, MSc, MD, FRCPE, FRCP, | CeSHHAR  Liverpool School of Tropical Medicine | Lead technical investigator and coordinates design, implementation & reporting on protocol. | [frances.cowan@lstmed.ac.uk](mailto:frances.cowan@lstmed.ac.uk) |
| Co-Principal Investigator | Professor James Hargreaves | PhD, MSc, BSc | London School of Hygiene and Tropical Medicine | Lead the tracing study and provide  support in all aspects of statistical analysis, qualitative data collection and process evaluation relating to Research Questions 1-4. | [james.hargreaves@lshtm.ac.uk](mailto:james.hargreaves@lshtm.ac.uk) |
| Co-Investigator | Associate Professor Joanna Busza | Associate Professor, MSc  B.A. | London School of Hygiene and Tropical Medicine | Lead the process evaluation of the AMETHIST trial, with a focus on the design and application of qualitative methods in all 3 countries | [Joanna.busza@lshtm.ac.uk](mailto:Joanna.busza@lshtm.ac.uk) |
| Co-Principal Investigator | Professor Andrew Phillips | PhD, MSc, BSc | University College London | Modelling and cost effectiveness analysis, design of trial and tracing study | [andrew.phillips@ucl.ac.uk](mailto:andrew.phillips@ucl.ac.uk) |
| Co-Investigator | Dr Loveleen Bansi Matharu | PhD, MSc, BSc? | University College London | Support in aspects of the Modelling | [l.bansi-matharu@ucl.ac.uk](mailto:l.bansi-matharu@ucl.ac.uk) |
| Co-Principal investigator | Professor Paul Revill | PhD, MSc, BSc | University of York | Advise and contribute in the design of cost effectiveness analysis and modelling | [paul.revill@york.ac.uk](mailto:paul.revill@york.ac.uk) |
| Co-Principal Investigator | Dr Fortunate Machingura | PhD, MPOS, CertPH, BSc | CeSHHAR Zimbabwe | implement the AMETHIST trial and all field work relating to Research Questions 1-3 in Zimbabwe and support Prof Frances Cowan to coordinate design, implement & reporting on the protocol | [fmachingura@ceshhar.co.zw](mailto:fmachingura@ceshhar.co.zw) |
| Co-Investigator | Mr Amon Mpofu | MSc, MBA, DipHE, CertEHT, BA | National AIDS Council Zimbabwe | Contribute as Co-I in Zimbabwe across research areas | [ampofu@nac.org.zw](mailto:ampofu@nac.org.zw) |
| Co-Investigator | Dr Owen Mugurungi | MD Dip GU Medicine MSc | Ministry of Health and Child Care | Contribute as Co-I in Zimbabwe across research areas | atp.director@ymail.com |

## Role of Participating institutions

- **Liverpool School of Tropical Medicine (LSTM):** The LSTM is the lead institution with responsibility for scientific and financial oversight and governance of the AMETHIST Consortium.
- **CeSHHAR Zimbabwe** is the Zimbabwe research institution, will implement the AMETHIST trial and all field work relating to Research Questions 1-3 in Zimbabwe.
- **London School of Hygiene and Tropical Medicine (LSHTM)** will lead the Data Technical Working Group and support all aspects of statistical analysis, qualitative data collection and process evaluation relating to Research Questions 1-4.
- **University College London (UCL)** will lead all aspects of the Modelling (Research Questions 3 and 4).
- **Africa Health Research Institute (AHRI)** will lead all aspects of the research in South Africa (Research Question 4)
- **Malawi Liverpool Wellcome Trust Clinical Research Programme (MLW**) will lead all aspects of the research in Malawi (Research Question 4)
- **University of York** will advise on cost effectiveness modelling (Research Questions 3 and 4).
- **National AIDS Council** will advise on national direction, strategy and policy convening in Zimbabwe and where needed.

## Sponsoring Institution

Liverpool School of Tropical Medicine.

## AMETHIST trial overview

The impact of an intensified and differentiated needs-based Zimbabwe Sister’s programme (AMETHIST) will be evaluated in a pragmatic cluster randomised trial that will provide evidence to inform health policy. Twenty-two Sisters clinic sites were randomised in January 2019 using restricted randomisation to the intensified programme or usual care. Outcomes will be measured after 24 months, using respondent driven sampling surveys conducted in all 22 sites, coupled with an in-depth process evaluation and programme data analysis.

The overall goal of the AMETHIST trial is to determine whether it is possible to reduce the proportion of sex workers who are HIV negative and are not on PrEP and who report condomless sex in the past month (i.e. are at risk of HIV acquisition) OR who are HIV positive and have Viral Load > 1,000copies/µl and who report condomless sex in the past month (i.e. who are at risk of HIV transmission) using an adapted microplanning intervention to increase uptake to and optimal use of HIV prevention and treatment among female sex workers (FSW).

***Figure 1: AMETHIST trial overview***


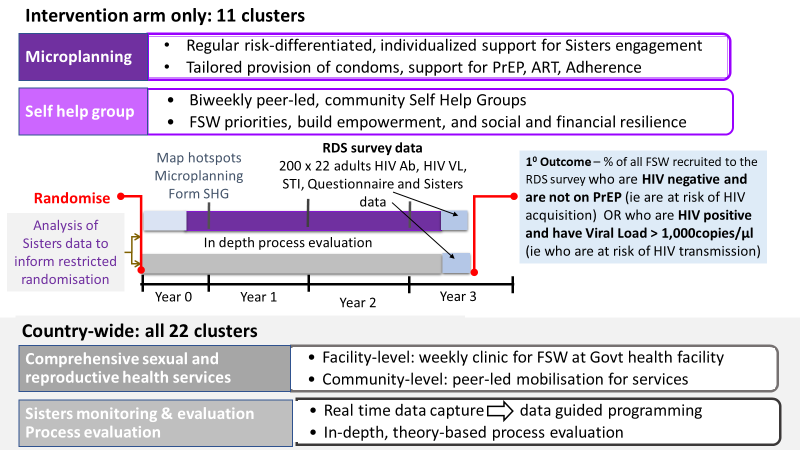


## Specific trial objectives

1. To determine if AMETHIST intervention (microplanning supported by formation of self-help groups) can be implemented at scale with sufficient intensity and coverage to reduce the proportion of sex workers at risk of HIV acquisition or transmission
2. To determine whether the AMETHIST intervention builds community empowerment

## Theory of Change

***Figure 2:Theory of change framework***

Our intervention will intensify sex work programming using a two-pronged approach as shown in Figure 4. Firstly, we will introduce microplanning to optimise coverage and provide risk differentiated community-based support for FSW to engage with prevention and care services. Secondly, we will support formation of self-help-groups to increase problem solving and skills, strengthen resilience and build social cohesion. Over time, as trust and empowerment grow, self-help groups have the potential to coalesce to form site-based sex worker community-based organisations and will have the potential to act as platforms for a range of public health interventions for this disenfranchised and vulnerable group and their families.

## Trial Endpoints

### Primary Endpoint

Primary endpoint data will be collected through respondent driven sampling surveys conducted at all 22 sites after 24 months. The primary end point is a composite endpoint to capture intervention impact among both HIV negative and HIV positive FSW.

% of all FSW recruited to RDS survey who are HIV negative and who are not taking PrEP and report condomless sex in the past month (i.e. at risk of HIV acquisition) OR who are HIV positive and have Viral Load > 1,000copies/µl and report condomless sex in the past month (i.e. who are at risk of HIV transmission).

### Secondary Endpoints

1. % of all FSW recruited to the RDS survey who are HIV negative and who are not taking PrEP (i.e. at risk of HIV acquisition) OR who are HIV positive and have Viral Load > 1,000copies/µl (i.e. who are at risk of HIV transmission).
2. It is planned to verify self-reports of consistent condom use in a sub-set of women through i) absence of sexually transmitted infection on vaginal swab collected during the RDS survey (Neisseria gonorrhoea, Chlamydia trachomatis and Trichomonas vaginalis) ii) absence of PSA/Y chromosome on vaginal swab collected during the RDS survey. (see secondary endpoint (vii) below.
3. % of all FSW recruited to the RDS survey who have had contact with the Sisters programme and who are HIV negative and who are not taking PrEP and report condomless sex in the past month (i.e. at risk of HIV acquisition) OR who are HIV positive and have Viral Load > 1,000copies/µl and report having condomless sex in the past month (i.e. who are at risk of HIV transmission).
4. % of FSW who enrolled in AMETHIST intervention compared with individually matched controls who are either HIV negative and  who are not taking PrEP and report condomless sex in the past month (i.e. at risk of HIV acquisition) OR who are HIV positive and have Viral Load > 1,000copies/µl and report having condomless sex in the past month (i.e. who are at risk of HIV transmission).
5. Proportion of HIV-infected women who are infectious (VL>1000 copies)
6. Proportion of those taking ART who have viral load >1000 and have drug resistance to their current regimen
7. Self-reported quality of life, psychological health and functioning
8. Proportion of FSWs reporting always using condoms with clients in last month who have evidence of Y chromosome/PSA antigen in vaginal specimen
9. Proportion of FSW offered, initiating and, where appropriate, adhering to/participating in:
   1. HIV testing, including quarterly repeat testing programmes for HIV-negatives
   2. Quarterly clinical assessment
   3. Pre-exposure prophylaxis for HIV-negatives
   4. Antiretroviral therapy for HIV positives
10. Proportion of FSWs who know HIV status (ie are diagnosed HIV positive or were tested HIV negative in last 6 months)
11. Perceived levels of peer support
12. Acceptability and perceived quality of services
13. Proportion lost to follow up (ie who have attended Sisters programme but last visit was > 12 months ago).

### Cost Analysis Endpoints

- 1. Total/overall programme economic cost (Sisters alone or Sisters plus AMETHIST intervention - is there possibility of costing no programme?)
  2. Dividing total programme costs by output indicators
     1. Average costs per person reached/ contact (Sisters alone or Sisters plus AMETHIST intervention)
     2. Average costs per person per service
  3. Variation by service delivery sites & over time (program maturity)
     1. Assess whether total costs, cost profiles and unit costs evolve over time due to programme learning effects and as staff get more efficient at their roles.
     2. Longitudinal nature of AMETHIST provides an opportunity for econometric analysis of cost determinants.
  4. Key cost contributors & other cost factors (program management, service delivery inputs, location)

### Cost effectiveness endpoints

The cost-effectiveness of our intervention package will be evaluated, by use of modelling to infer the reduction in DALYs incurred in the whole adult population as a result of the intervention, together with the difference in costs (including all HIV related costs in the population as a whole), taking into account the potential reduction in direct and indirect HIV acquisition and transmission occurring as a result transactional sex due to the reduction in the proportion of sex workers who are either infectious or at risk of acquisition.

### Process evaluation endpoints

To document implementation of the intervention, understand its mechanisms of action, and contribute to interpretation of trial results, a comprehensive process evaluation will be conducted throughout the trial period (see Annex 5 and 6).

#### Indicators of implementation

**Microplanning**

1. Number of empowerment workers hired and trained in microplanning
2. The number and completeness of hotspot mapping, identification of FSW & validation occurring at 6 monthly intervals in each site
3. Weekly individual supervision meeting and monthly collective support meeting with all empowerment workers

**Self-help Groups (SHGs)**

1. Number of empowerment workers trained to initiate and support 2x/month SHG in 6-month cycles
2. Number (%) of empowerment workers retained in post over 2 years
3. Number of outreach workers employed to oversee and troubleshoot SHG programme
4. Biweekly supervision of all SHG meetings by outreach workers for first three months with monthly supervision from 3-12 months

#### Determinants of target behaviours

| - % of all FSW in identified hotspots entered into hotspot diary | - % micro planned FSW referred to & taking up Sisters services |
| --- | --- |
| - % of high, medium and low risk FSW (see Table 3 below) tracked according to risk status | - Number of self-help groups established (FSW/SHG = 15) |
| - Number of self-help groups continuing to run with at least 70% of original attendees over 6, 12 and 18 months | - % of FSW registered in hotspot diaries enrolled in and attending self-help groups |
| - Levels of acceptability of microplanning and SHG to EW and FSW peers | - % SHG members taking up Sisters services |
| - Perceptions of group trust by SHG & EW | - Examples of SHG action/initiatives |
| - Changes in financial literacy and resilience (Management and saving of money improved) | - Changes in perceived mental well-being by SHG members |
| - Changes in perceived social, material and practical support by SHG members | - Perception of peer norms regarding health-seeking |
| - Perceptions of social support among FSW | - Target behaviour |
| - % FSW attending Sisters clinics quarterly | - % of all FSW assessed quarterly for risk |
| - Levels of feasibility to deliver microplanning and SHG by CeSHHAR staff |  |

## Study location

This study will run through the Centre for Sexual Health and HIV Research (CeSHHAR), Zimbabwe which runs Zimbabwe’s National Sex Work Programme ‘Sisters with Voice’. Twenty-two sites which have Sisters FSW clinics running within them since at least 2014 will be randomised to the AMETHIST intervention or usual care. Randomisation will be restricted on a range of programme indicators collected from sites in 2017.

The 22 outreach sites to be included in the trial are: Juru, Murewa, Mutoko, Nyampanda, Karoi, Chinhoyi, Kariba, Magunje, Chirundu, Kadoma, Kwekwe, Zvishavane, Gwanda, Hwange, Lupane, Chipinge, Beitbridge, Ngundu, Chivhu, Marondera, Chiredzi, Rusape. (See Figure 5).

***Figure 3: AMETHIST trial sites***

***(Figure to be updated)***


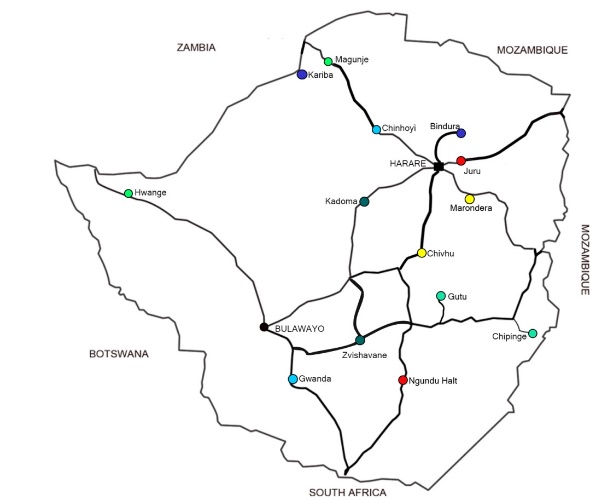


## Sisters – Zimbabwe’s National Sex Worker Programme

### Usual care

The Sisters with a Voice programme provides free condoms and contraception, provider-initiated HIV testing and counselling, HIV self-testing and counselling (and secondary distribution of self-test kits for partners), syndromic management of sexually transmitted infections, health education and legal advice. Additionally, clinics are providing long-acting reversible contraception (implant), referral for cervical cancer screening and on-site access to PrEP. Programme data are collected electronically in real-time. The programme is supported by peer educators who mobilise FSW to attend clinical services and encourage uptake of HIV testing and support referral of FSW for ART and PrEP as appropriate. The programme is run through dedicated Sisters clinics based within government primary care clinics in towns and along highways in Zimbabwe. Approximately 200-500 FSW are seen at each Sisters site annually. Women who test positive for HIV are referred to government services for HIV care/ART initiation. This set of WHO-guided standard services will form the comparison communities in this trial.

### AMETHIST Intervention (Adapted Microplanning to Eliminate Transmission of HIV In Sex Transactions)

The intensified AMETHIST intervention will provide usual care augmented by microplanning and establishment of self-help groups in intervention communities.

In AMETHIST intervention communities, FSW peer educators are trained to undertake microplanning and form and facilitate self-help groups. The specially trained peer educators are known as Empowerment Workers (EW). EW in each site, map geographic sex work ‘hotspots’ and an estimate of the total number of hotspots is made. In addition, an estimate of the total number of FSW working at each hotspot is made so that an estimate of the overall size of the sex work population in that site can be made. A validation of the hotspot mapping is made with a member of CeSHHAR staff. Hotspot mapping and validation is repeated every 6 months.

It is intended that each EW is responsible for microplanning around 50 FSW in a hotspot (larger hotspots may be allocated to more than one EW, one EW may look after more than one smaller hotspot). The number of EW trained and supported to manage each site is therefore dependant on the number of FSW working there (# EW at a site = total number of FSW working at a site/50).

Each EW is allocated a specific hotspot at which to work. There she enumerates all FSW working at her allocated hotspot, providing each FSW she identifies a Sisters ID number (or recording her Sisters ID number if the FSW has already attended the Sisters programme). She meets with each FSW in her hotspot on a regular basis (known as tracking). Once the EW worker has developed a relationship with an FSW, she asks her a few questions to ascertain how vulnerable she is using a simple score (see Table 3).

***Table 2: Risk Assessment tool***

EW aim to assess the vulnerability of all FSW in their hotspot every 3 months. Those FSW at highest risk are then tracked weekly, those at low risk monthly. At each microplanning contact, EW provide support, appropriate to individual FSW, and guided by data collected at their last contact e.g. revisiting previous discussions, reminding women about clinic appointments, providing her with condoms etc. Microplanning generates data for subsequent contacts; EW update the information they have on each FSW at each contact, analyse it, and work with their supervisors to plan which FSW to meet with and what should be the topics for discussion. Importantly microplanning is status neutral (i.e. the EW does not need to know or ask the HIV status of each FSW). EW encourage all FSW in their hotspot to be seen for clinical review every 3 months (where her need for HIV testing, ART initiation and or adherence support, PrEP initiation and or adherence support, contraception, STI screening and treatment etc) can be assessed.

In addition to microplanning, each EW invites some of the women in her ‘microplanning caseload’ to join a self-help group. Self Help groups comprise around 10-15 women which aim to meet twice a month. The EW facilitates the self-help group, initially with the help of a member of programme staff. FSW receive training on how to run a group, keep a register, manage money (including how to budget, save, open and manage a bank account) and keep minutes if required. Each self-help group is facilitated to identify their own priorities and receives support towards these. For example some groups set up child care facilities whereas others opt to set up a form of savings and lending scheme. Training is provided to support groups with their particular priorities. These self-help groups strengthen programme ownership and community empowerment, to reduce HIV risk and increase uptake of services among FSW (17) and among the general population of women(43). Over time, as trust and empowerment grow, self-help-groups have the potential to become platforms for a range of public health interventions for this disenfranchised and vulnerable group and their families.

### Supporting engagement with prevention and care activities as part of the Sisters programme

The Sisters programme has an electronic patient monitoring system programmed in DHIS2 into which all contacts with the programme are recorded including community outreach contacts and attendance at Sisters services. The microplanning app within this platform generates dashboards to guide the weekly and monthly supervision of empowerment workers and provides them with reminders of which of the FSW on their registers should be attending clinic visits. The programme is also able to alert programme staff to FSW who have missed their quarterly clinic appointment. All women (HIV positive and negative) who have consented to do so have detailed locator information collected so that they can be contacted with reminders prior to appointments or traced if they miss a clinic appointment. Defaulters include all FSW who fail to attend their quarterly clinic visit, HIV negative FSW on PrEP who fail to attend for prescription refill, and HIV positive women eligible for ART who default from their recommended clinical care.

## Trial Design

The trial is a cluster randomised controlled trial.

### Cluster definition

A cluster is defined as the SW ‘catchment’ population around a government health clinic where dedicated SW services are being delivered through the Sisters programme.

### Randomisation

Twenty two Sisters sites were randomised (1:1) to receive the enhanced microplanning intervention or standard care (randomisation conducted under MRCZ/E/206 approved protocol "Cluster Randomised Trial of Micro planning: An HIV Prevention and Care Differentiated Community Support Model for Female Sex Workers" in January 2019 at a public randomisation meeting hosted by National AIDS Council. To maximise transparency and buy-in from key stakeholders, NAC invited MoHCC, district representatives and representatives of female sex worker community from the 22 sites.

From June 2019, eleven sites have had microplanning and self-help groups introduced as a programme improvement in addition to the standard services offered by the Sisters programme. Routine programme monitoring data are being collected. The protocol MRCZ/E/206 will be merged with this protocol once approved.

Imbalance between arms was minimised by using restricted randomisation and matching of clusters using programme data from 2017 on site size, numbers of female sex workers seen at the programme, number of younger sex workers seen at the programme (aged 18-24), number of sex workers seen for the first time and number of young sex workers seen for the first time (aged 18-24).

### Measurement of primary and secondary endpoints

Endpoint data will be collected from the sources described below in Table 4.

***Table 3: Trial endpoints***

| **Primary Endpoint** | **Data Source** |
| --- | --- |
| % of all FSW recruited to RDS survey who are HIV negative and who are not taking PrEP (i.e. at risk of HIV acquisition) OR who are HIV positive and have Viral Load > 1,000copies/µl (i.e. who are at risk of HIV transmission). | Respondent driven sampling survey (RDS) |

| **Secondary Endpoints** | **Data Source(s)** |
| --- | --- |
| 1. % of all FSW who have been in contact with the Sisters programme who are HIV negative and who are not taking PrEP (i.e. at risk of HIV acquisition) OR who are HIV positive and have Viral Load > 1,000copies/µl (i.e. who are at risk of HIV transmission). | RDS + programme |
| 1. % of FSW who enrolled in AMETHIST intervention compared with individually matched controls who are HIV negative and who are not taking PrEP (i.e. at risk of HIV acquisition) OR who are HIV positive and have Viral Load > 1,000copies/µl (i.e. who are at risk of HIV transmission). | RDS |
| 1. Proportion of HIV-infected women who are infectious (VL>1000copies/uL) | RDS |
| 1. Proportion of those taking ART who have viral load >1000 who have drug resistance | RDS |
| 1. Self-reported quality of life, psychological health and functioning | RDS |
| 1. Proportion of FSWs reporting always using condoms with clients in last month who have laboratory evidence of STI | RDS |
| 1. Proportion of FSWs reporting always using condoms with clients in last month who have evidence of Y chromosome/PSA antigen in vaginal specimen | RDS |
| 1. Proportion of sex workers offered, initiating and, where appropriate, adhering to/participating in: |  |
| - 1. HIV testing, including quarterly repeat testing programmes for HIV-negatives | RDS, Programme data |
| - 1. Quarterly clinical assessment | RDS, Programme data |
| - 1. Pre-exposure prophylaxis for HIV-negatives | RDS, Programme data |
| - 1. Antiretroviral therapy for HIV positives | RDS, Programme data |
| 1. Proportion of SWs who know HIV status (i.e. are diagnosed HIV positive or were tested HIV negative in last 6 months) | Programme data |
| 1. Proportion lost to follow up. | Programme data |

| **Cost effectiveness Endpoints** | **Data Source** |
| --- | --- |
| - 1. Total/overall programme economic cost (Sisters alone or Sisters plus AMETHIST intervention - is there possibility of costing no programme?)   2. Dividing total programme costs by output indicators      1. Average costs per person reached/ contact (Sisters alone or Sisters plus AMETHIST intervention)      2. Average costs per person per service   3. Variation by service delivery sites & over time (program maturity)      1. Assess whether total costs, cost profiles and unit costs evolve over time due to programme learning effects and as staff get more efficient at their roles.      2. Longitudinal nature of AMETHIST provides an opportunity for econometric analysis of cost determinants.   4. Key cost contributors & other cost factors (program management, service delivery inputs, location) | Costing data |

### Respondent Driven Sampling Surveys

Respondent Driven Sampling (RDS) is an adaptation of chain referral sampling, where initial “seeds” are selected to represent a range of characteristics (e.g. diversity in age, location, type of sex work). After completing the questionnaire, each seed starts a recruitment “chain” by recruiting a specified number of peers into the survey. Each subsequent respondent is further provided with coupons to give to several peers to refer them into the study; participants receive financial compensation for completing the survey themselves as well as for each of their recruits who participates.

RDS has been shown to reduce sampling bias and to improve representativeness of “hard-to-reach” populations by limiting the number of referrals any one respondent can have (thus creating “deep” rather than “wide” sample networks), and through the use of specific statistical techniques that weight the recruitment patterns in a way to balance out inconsistent recruitment.

Sex workers in each trial community will be surveyed after 24 months of intervention using RDS in order to assess 'population level' effects of the program. Surveys will aim to include 200 women per cluster in each of the 22 clusters.

#### Inclusion and Exclusion Criteria for Survey

All clusters will be included in the survey. In each cluster, seeds will be selected following 2-3 days of geographic and social mapping of sex work in each location. Mapping will assist in understanding the local context adequately to identify specific criteria for seeds in each site and how many should be selected. Geographic and social mapping includes informal discussions with trained peer educators, healthcare staff, and community informants. Research staff involved in mapping and seed selection will not be programme staff and seeds will be selected independently of the programme.

**Inclusion criteria:**

- Age 18 or older
- Currently working as a sex worker (has exchanged sex for money in the past 30 days)
- Living or working in the study site (for at least 1 month)

**Exclusion criteria**

- Under 18
- Already participated in the current round of survey
- Visiting the study site temporarily (for less than 1 month)
- Not currently working as a sex worker (verification questions will be used to prevent fraudulent participation, such as about how sex work is organised in the area and the cost of different sex acts)

Eligibility will be determined by research staff on arrival to the RDS survey site. Research staff will check that each recruit reporting for enrolment has a valid referral coupon from a previous survey participant (except for seeds), meets the eligibility criteria and can answer the verification questions, understands the purposes of the study and can provide informed consent. Research staff will review the consent form with the participant. If the participant acknowledges full understanding of her participation in the survey, she will be enrolled. The RDS survey instrument itself further contains questions that will check eligibility (such as current age and whether or not she has sold sex in the previous 30 days).

#### Survey design, methods, and procedures

**Interview Sites**

In each site following mapping the research team identify a central location where women report they would be happy to go and take part in the study. This usually includes community centres or halls. There is no identifying signage. It is selected for its accessibility to local sex workers, relative discreetness, and availability of enough space to arrange a waiting room (for screening and payments for recruiters) as well as a private room for behavioural interviews and blood sample collection. Informal interviews conducted during mapping will assist in determining suitable location in each of the study sites.

**Recruitment**

Between 6 and 10 seeds will be selected to reflect salient SW socio-demographic characteristics, as identified through mapping in each cluster. Seeds are selected following a mapping exercise conducted by research rather than programme staff in each site. Seeds will complete all study procedures and will then be provided with two numbered coupons to recruit the first wave of peer participants.

**Multiplicity**

Duplication of respondents is a concern in any survey, but may be more likely when using RDS if: (a) sex workers do not understand the process of peer recruitment and refer the same peers more than once or refer reciprocally, or, (b) sex workers attempt to return using the same coupon in order to redeem the compensation payment more than once. Efforts to avoid duplicate participation will be made. First, coupons will be collected by survey staff when brought in by recruits; similarly, the portion of the coupon retained by the recruiter will be collected by staff when secondary compensation is made. Furthermore, aa study “code” will be compiled made up of the respondent’s initials and birthday and recorded for cross-checking against new study recruits. This is checked in real time electronically against study codes already generated at that site so that potential duplicates can be easily identified if they report the same initials and date of birth.

**Incentives**

Participants (including seeds) will receive compensation of equivalent of US$5 for completing the interview (primary incentive) and further compensation of equivalent of US$2 (secondary incentive) for each of their peers whom they recruit and who complete the survey (so up to a total of US$9 per participant). Recruiters will receive their secondary incentives as long as her recruit presents a coupon, fulfils the eligibility criteria, and enrols in the survey. The amount of compensation was set to be within the requirements of the MRCZ and are designed to be high enough to compensate for potential loss of 1 client during study participation, but not so high as to encourage significant fraudulent enrolment.

**Tools**

The RDS survey will consist of two components, a behavioural questionnaire self-administered on a tablet using an audio computer assisted survey instrument (ACASI). Additionally, a finger prick blood sample will be taken for HIV testing, syphilis testing, HIV viral load testing, drug resistance testing and ART level testing (as appropriate). In addition, all participants will be asked to provide a self-administered vaginal swab to be tested for sexually transmitted infections and biomarker of condomless sex. The questionnaire will collect basic socio-demographic information (age, marital status, education, etc.), economic characteristics, sexual behaviour, alcohol use, psychological health, quality of life, physical health, past history of sexually transmitted infections, sexual and social networks, social capital, utilisation of services including HIV testing, ART, PMTCT and family planning. Questions also be asked information related to past or present experience of psychical or sexual violence with clients and the police. The questionnaire will also include specific RDS network questions to allow for RDS analysis of recruitment chains. Of note CeSHHAR has a referral plan to support any participant who become emotionally distressed during the interview process (Annex 7). The questionnaire will be developed in English and then translated into local languages (Shona and Ndebele). The questionnaire will be labelled with a unique Participant ID number (PTID) and linked to the biological specimen through a CAPI log. The CAPI log will link an individual’s PTID with their biological specimen labels which will use a unique non-human readable bar code.

**Laboratory methods**

All participants will have a finger prick blood sample taken for rapid HIV testing in the field. Samples will be tested according to the Zimbabwe National HIV testing algorithm with samples tested in series (see Annex 7 – Chapter 10). The syphilis sample will be tested using CHEMBIO DPP® SYPHILIS SCREEN and CONFIRM PoC test, a near patient test that tests for both Rapid Plasma Reagin (RPR) and Treponema Pallidum Haemagglutination Assay (TPHA) in a single test in 20 minutes (Annex 7 – Chapter 12). Results of HIV and Syphilis rapid tests will be returned to all participants on site.

In addition, all women will have two dried blood spot samples collected for HIV viral load testing and potential ART and drug resistance testing at the Flow Cytometry Laboratory. Results of viral load tests will be made available to women within 4 weeks of the survey. A letter explaining viral load results will be provided for women to take to their ART provider.

A point of care urine (POC) assay was recently developed to measure TFV levels in urine.(48) The POC lateral flow assay has a threshold of 1500ng/mL consistent with dosing in the last 4-7 days based on a directly observed therapy study, is being developed in collaboration with Alere Diagnostics, and will cost approximately $3. Approximately 2-3 minutes after 3-4 drops of urine are placed on the urine test strip, a “control” line should appear. An additional “tenofovir test line” will appear only if a participant has TFV levels <1500 ng/mL indicative of no recent PrEP dosing.(49)

All participants will be asked to collect a self-administered vaginal sample. All STI samples will be transported to Flow Cytometry Laboratory within 48 hours at 4oC. Samples will be tested by the Flow Cytometry Laboratory using Xpert® TV and Xpert® CT/NG. Results of STI tests will be made available to women through the Sisters clinic within 4 weeks of the survey and free treatment will be made available and contact tracing of sexual partners offered. If women with evidence of an STI fail to return for their STI results, we will phone up to three times to encourage them to attend the clinic for treatment. Additionally, vaginal samples will be tested for biomarker evidence of condomless sex (PSA antigen or Y Chromosome).

## Intervention monitoring and process evaluation framework

The AMETHIST pragmatic cluster randomised control trial will test an enhanced package of community-based outreach and mobilization activities on population level reductions risk of HIV acquisition and transmission among female sex workers (FSW). To document implementation of the intervention, understand its mechanisms of action, and contribute to interpretation of trial results, a comprehensive process evaluation will be conducted throughout the trial period.

### How will the AMETHIST intervention lead to positive change?

The Project Trajectory (Figure 6) illustrates how integrating Microplanning and Self-Help Groups into the Sisters with a Voice national sex worker prevention and care programme is hypothesised to lead to its intended outcomes. Empowerment Workers will systematically map FSW in identified hotspots, conduct quarterly risk assessments, and use resulting evidence to provide risk-differentiated support for engaging FSW in prevention and care services. In parallel, FSW will be encouraged to join Self Help Groups facilitated by EW to build networks of support, strengthen individual and group-based problem-solving skills, and increase FSW capacity to work together to identify shared priorities and collectively act to address these.

Assuming EW are adequately trained, supported and supervised, their delivery of MP and SHG should create an enabling environment in which FSW at each site are reached according to need, equipped to address determinants of their vulnerability, and actively engaged in care. In turn, enhanced resilience, community support and service use will contribute to reducing FSW risks of acquiring and transmitting HIV, as well as improve their broader physical and mental health.

### Assessing Implementation of AMETHIST

For progress along this proposed trajectory to occur, however, its implied logic must be sound, intervention components must be delivered as intended and with suitable quality, and the local context must allow sufficient feasibility and acceptability for the designed effects to occur. In line with the MRC Guidelines for Process Evaluations, we have developed a framework to document and assess the following (1) Implementation (what was delivered and to what extent did this adhere to planned design?); (2) Mechanisms of action (how did FSW engage with different aspects of the intervention and what effects did this have on their behaviour and its determinants?); and (3) Context (how did local political, social, economic and health systems conditions affect intervention feasibility, acceptability and effects?).

The methods used to capture project implementation at each level are illustrated in the Evaluation Framework (Figure 6). Both quantitative and qualitative data collection will monitor delivery and uptake of each programme component over time, examining ease of delivery, varying rates of participation by FSW and their experiences, perceptions and satisfaction levels. Changes, omissions, and delays from the intervention design will be explored from the perspectives of implementing staff, key informants, and other stakeholders. Site-specific and national-level contextual influences on how the intervention is delivered, received, and able to effect changes along the hypothesised pathway will be captured through interviews and real-time “project diaries.” Specific modules within RDS surveys will complement participatory and qualitative means of assessing interim social processes such as increased social cohesion, resilience, and collective capacity.

## Costing analysis

The costing objective is to measure and analyse economic costs of scaling up the integrated model of peer-led microplanning supported by self-help-groups for FSW in Zimbabwe. Economic costs include financial costs representing actual expenditure on goods and services and volunteer time and/or donated goods for which there were no financial transactions.

1. Prospective costing study
   1. The prospective nature of AMETHIST provides an opportunity for us to examine costs over time as it is not limited by the short nature of most studies. Specifically, we will assess:
      1. Assess the changing cost structure of HIV prevention with intervention roll-out.
      2. Cost variation with programme evolution and scale.
2. Top down costing approach.
   1. Comprehensive access to data (CeSHHAR expenditures, time sheets, M&E etc) to facilitate allocation of program expenditures to sites and then to activities
   2. Costs will be disaggregated and categorised by activities and input type.
      1. Startup, capital and recurrent costs
   3. Approach captures total cost better though less precise for disaggregating costs at the activity level.
   4. First allocating out specific expenditures that could be clearly tracked to sites, then by M&E outputs such as persons reached, distances etc
   5. All expenses prior to first person reached by AMETHIST programme will be treated as start-up costs and together with capital costs annualised at 3% discount rate
3. A bottom up approach will supplement costing (more site level data collection) to ensure robustness of cost allocation
   1. SaPPHIRE data for baseline cost analysis in all 22 clusters
   2. Includes interviews with site program staff.
   3. Time and motion analysis
4. Cost data analysis
   1. Data will be entered and analysed in a Microsoft Excel spreadsheet

## Monitoring and process evaluation data collection

### Checklists

A prospective record should be maintained of if/when planned activities (as per protocol) were implemented.

### Staff and training records

Routine programme documentation on start-up activities of the project, including the number of staff hired and deployed, and how many attended all relevant trainings. These will be reviewed annually and at the end of the project if staff retention/performance considered to be an issue affecting the delivery of intervention components.

### Microplanning and Self-Help Group records

Empowerment workers will be given tablet computers on which to record all contact with peers as outlined in the microplanning handbook (see Annex 5.1 – Annex 5.3).

### RDS surveys

The RDS survey questionnaire will include some process endpoints related to intervention uptake in the intervention arm.

#### Qualitative data

- **Semi-structured interviews with clinic staff –** Semi-structured interviews with staff at intervention clinics (including any staff who have left the programme) will be conducted to elicit perceptions of the feasibility and acceptability of the intervention and their own levels of satisfaction and perspectives on its quality. Specific issues will be probed, such as ease of delivering the enhanced package, particularly challenges faced in increasing community mobilisation efforts through microplanning and self-help groups, promoting quarterly clinic visits, timely uptake of treatment, and introduction of PrEP.
- **In-depth interviews with FSW –** In-depth interviews with FSW will be conducted at three intervals across the project (baseline, mid-implementation, end) in both enhanced intervention and comparison sites. SWs will be purposively selected for specific attributes such as high or low levels of engagement in activities (in intervention areas), experience as peer educators or SW care supporters, users and non-users of clinical services for SW, those eligible and ineligible for treatment, and different ages and types of sex work. Interviews will explore perceptions of community cohesion and social support among SW, perceptions of clinical and other available services, and positive and negative experiences of the intervention (or reasons for choosing to participate or not) in intervention sites.
- **Semi structured interviews with key stakeholders –** Semi structured interviews with key stakeholders, including CeSHHAR management and staff, and representatives of other partner organisations such as MoHCC, UNFPA, PSI and services to which referrals were made.
- **Project diary –** A calendar or diary will be kept by a member of the CeSHHAR research team to record any events (particularly external) that may affect ability of the intervention to be delivered as planned. Elections, police crackdowns, local festivities, other health promotion campaigns should all be noted, with a description of their activities and effect. Internal events that have bearing on implementation (such as increased funding, or break-down of a project vehicle) will also be noted to help contextualise other information collected.

## Data management

CeSHHAR Zimbabwe will be the data coordinating centre. This unit is headed by an experienced data manager. Data will be cleaned, entered, analysed and safely stored there.

Data management and security standards will be equivalent for routine programme data and data collection in RDS surveys, though there will be significant differences in the way data are collected. For RDS, data will be collected using ACASI with assistance from a trained female interviewer. Data validity checks will be built into the ACASI platform. In the field, data will be backed up daily onto a memory stick and/or into “cloud storage”. Laptop computers on which any data may be stored will be kept in locked storage at all times. In the field, consent forms are kept in locked trunks and returned to the CESHHAR offices each week. Field teams will return to Harare at the end of each week to download data into a password protected database accessible only to the project data manager and named study personnel, on a central computer. This will be backed up daily. The consent forms are kept separately from survey data. In Harare consent forms will be stored in a locked room at CeSHHAR offices.

For programme data, one hard-copy file linking participants’ names with ID numbers, and contact/locator information will be maintained by the Project Coordinator and stored in a secure locked cabinet separate from participant data. Other hard-copy data will be stored separately in participant files and locked in a file cabinet located in a secure room accessible only to key study personnel. Participants will be asked to provide written informed consent for participation in the RDS survey and qualitative interviews (see Annex 3.1 – 3.3 and Annex 4.1 -4.3).

## Statistical considerations

#### Sample Size Calculations

The sample size is based on pragmatic considerations. Outcomes will be assessed after 2 years of implementation through respondent driven sampling surveys (n=200 per cluster) conducted within each cluster. Analyses will be adjusted accordingly. With 22 clusters, we have 80% power to detect a 30% difference in % FSW who are at risk of HIV acquisition/transmission between the intervention and control arms. Our estimate that 30% of FSW currently meet our primary outcome definition across the sites is based on data from the SAPPH-IRe trial control arm.

In Table 5 we show power calculations, considering different baseline prevalence of the outcome and effect sizes. The number of clusters and individuals per site is fixed (at 22 and 200) as it is not feasible to conduct a larger trial in Zimbabwe.

***Table 4: Power calculations***

***
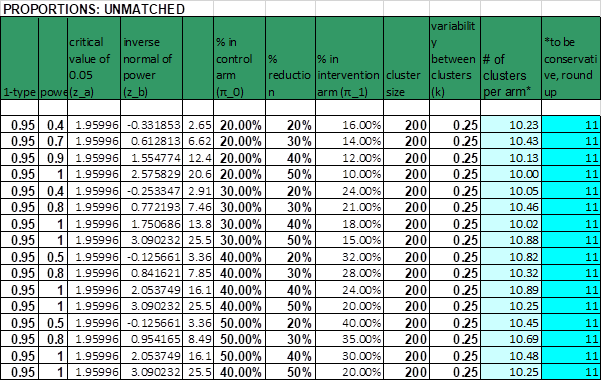
***

#### Statistical Analysis

We will first conduct descriptive and RDS-diagnostic analyses to understand the population recruited through the RDS process. Our primary analysis will be based on the intention-to-treat principle. To account for clustering in the data, data from individuals will be summarised for each cluster. We will calculate cluster summary means using the RDS-2 methodology developed by Volz and Heckathorn in which individuals are weighted with proportion to 1/self-reported out-degree2.

To estimate network degree, we will use the number of sex workers a participant reported knowing who were at least, 18 years old, lived at the site, and who the participant would consider recruiting to the study. We will drop the seed responses from the analysis and weight the results for each woman in each site by the inverse of her network size (ie, the number of other women thatshe could have recruited).

For the unadjusted analysis, we will fit a linear regression model on the RDS-weighted cluster summaries at endline, with a treatment dummy, and RDS-weighted cluster summaries at baseline as regressors. We will use the p-value and confidence intervals for the coefficient of the treatment dummy.

To address the possibility of confounding from chance imbalance, we will conduct an adjusted analysis using the ‘two steps’ method of Hayes and Moulton3. Age is specified a priori and will be included as a potential confounder that could affect the outcome but is not on the causal pathway. Other factors will be included if they appeared imbalanced post-randomisation. We will use descriptive statistics to assess whether there was balance across the two arms in key socio-demographic and potentially confounding variables at endline RDS surveys. Results will inform those variables to be adjusted for in the adjusted analysis, with particular attention paid to variables that are known a priori to be associated with the endpoints of interest. We will also explore and report response rates across the arms. Full details of the adjustment strategy will be included in a statistical analysis plan finalized before the data are analysed.

To generate adjusted, RDS-weighted analysis, we will fit an individual-level logistic regression model with the primary outcome as the dependent variable, and age and any other confounders as independent variables.

This model will be used to generate predicted probabilities for each woman. For each cluster, we will calculate the weighted arithmetic mean of the predicted probabilities, weighted by the inverse of the network degree to account for the respondent driven sampling. The weighted mean of the predicted probabilities will be interpreted as the RDS-II-weighted predicted prevalence for each cluster.

We will calculate the ‘residual’ risk difference (RD) by subtracting the predicted prevalence from the RDS-II weighted observed prevalence in each cluster. We will fit a linear regression model on the residuals to calculate the adjusted RD. The strength of evidence against the null will be assessed using an unpaired t-test.

## Ethical considerations

This protocol will be subject to review and approval by institutional review boards at all participating institutions, including those in Zimbabwe (the Medical Research Council of Zimbabwe), Liverpool School of Tropical Medicine, University College London and London School of Hygiene and Tropical Medicine. This will include, according to each IRB’s requirements, approval prior to the initiation of research, on-going adverse event monitoring, periodic review, and final study reporting. The protocol has been approved by the Ministry of Health and Child Care and the National AIDS Council who work in partnership with Sisters program to implement this study.

Of note randomisation of sites for the trial (Research Question 2) took place prior to research funding becoming available and a protocol entitle “Cluster Randomised Trial of Microplanning: An HIV Prevention and Care Differentiated Community Support Model for Female Sex Workers" - was submitted to and approved by MRCZ (MRCZ/E/206). This protocol was to randomise sites to AMETHIST intervention or standard of care and measure outcomes using programme data only. No research data were to be (or have been) collected under protocol MRCZ/E/206.

### Participant informed consent and remuneration

Survey participants will be recruited under good clinical practice by experienced and female researchers trained and certified in Ethics, Good Clinical Practice and Safeguarding. Written informed consent (Annex 3), for survey participation will be conducted. Participants will be provided with information about the research and study procedures by a trained female interviewer and will have the opportunity to ask questions as part of the informed consent process. Standard guidelines for illiterate participants will be followed and are detailed in the SOP.

Women who participate in the RDS surveys will be recruited through RDS survey techniques, which include recruiting 6-10 initial seeds at each site in both the intervention and control communities (see Annex 7). Participants will receive financial compensation of US$5 for completing the survey themselves as well as US$2 for each of their recruits who participates. SW, health care workers and other stakeholders who are interviewed as part of process evaluation will also be recompensed with the standard US$5. (see Annex 10 and 11)

### Confidentiality

The research team will ensure that all research data collected are numbered with a unique ID and not named. A link log which links the unique ID to name will be kept by the project coordinator in Harare in a locked and secure place, separate from the questionnaire/interview data. Names, addresses and other identifying information will be required at the clinic sites for follow up purposes, and will be kept separately from questionnaire and laboratory data. Only the site coordinator, data manager and principal investigator will have access to this information. Laboratory and questionnaire data will be linked using an individual's unique study ID number.

All study staff will undergo GCP and ethics training. All people working with CeSHHAR Zimbabwe sign a confidentiality agreement; they have strict confidentiality procedures in place (see Annex 2).

### AMETHIST Trial Data and Safety Monitoring Board

A Data and Safety Monitoring Board (DSMB) will be established and will meet at the outset of the trial using Skype/ZOOM, and then semi-annually. The DSMB will approve the overall protocol, assessments, and consent forms. The DSMB will receive all reports of adverse events, as will the IRBs overseeing this study. Meetings of the DSMB can be scheduled, as needed, to discuss and resolve AE issues. The DSMB will review semi-annually all accrued data to assess that study objectives are being met, and to ensure that benefit exceeds harm. The DSMB will see and approve the Statistical Analysis Plan prior to endline analysis. DSMB membership Professor Katherine Fielding LSHTM, Professor Helen Ward Imperial College London, Professor Nancy Padian University of California San Francisco, plus two Zimbabwe sex workers (Tsitsi Ndabambi and Audrey Mushonga).

### AMETHIST Community Advisory Board

The trial will establish a community advisory board (CAB) comprising one SW and one community stakeholder from each of the trial communities. The mandate of the SW Community Advisory Board is to strongly link the trial steering committee and investigators to the SW community, ensure transparent and full communication between trial and the community it serves, and to ensure that the trial research findings have a maximum impact on the communities from which the information is drawn. The CAB functions in an advisory capacity to the trial steering committee, which will be endeavoured to respond appropriately to the issues raised. The CAB will assist trial staff in developing culturally and linguistically appropriate materials for study participants such as study specific fact sheets and informed consent forms.

### Trial modification and discontinuation

The study may be modified or discontinued at any time by the IRBs as part of their duties to ensure that research participants are protected. No interim analyses are planned.

### Protocol deviations and exceptions

The investigator should not implement any deviation from, or changes of, the protocol without prior review and documented approval from the Ethical Committee of an amendment, except where necessary to eliminate an immediate hazard(s) to trial subjects. The investigator should document and explain any deviation from the approved protocol and to file waivers received from the MRCZ, if applicable.

***Figure 4: AMETHIST Trial Process Evaluation framework***
